# Explainable age prediction using a transformer trained on diverse global electrocardiograms

**DOI:** 10.1093/ehjdh/ztag149

**Published:** 2026-09-23

**Authors:** Laurenz Berger, Kristina Preuer, Christoph Gross, Max Haberbusch, Mohammad Azizzadeh, Noemi Pavo, Marie-Kathrin Breyer, Francesco Moscato

**Affiliations:** Center for Medical Physics and Biomedical Engineering, Medical University of Vienna, Währinger Gürtel 18-20, AKH-4L, A-1090 Vienna, Austria; Ludwig Boltzmann Institute for Cardiovascular Research, Institute of Vascular Biology and Thrombosis Research, Schwarzspanierstraße 17 H, A-1090 Vienna, Austria; Center for Medical Physics and Biomedical Engineering, Medical University of Vienna, Währinger Gürtel 18-20, AKH-4L, A-1090 Vienna, Austria; Institute of Biomedical Mechatronics, Johannes Kepler University Linz, Linz, Austria; Ludwig Boltzmann Institute for Lung Health, Site Penzing of Clinic Ottakring, Sanatoriumstraße 2, A-1140 Vienna, Austria; Faculty of Medicine, Sigmund Freud Private University, Vienna, Austria; Center for Medical Physics and Biomedical Engineering, Medical University of Vienna, Währinger Gürtel 18-20, AKH-4L, A-1090 Vienna, Austria; Ludwig Boltzmann Institute for Cardiovascular Research, Institute of Vascular Biology and Thrombosis Research, Schwarzspanierstraße 17 H, A-1090 Vienna, Austria; Ludwig Boltzmann Institute for Lung Health, Site Penzing of Clinic Ottakring, Sanatoriumstraße 2, A-1140 Vienna, Austria; Faculty of Medicine, Sigmund Freud Private University, Vienna, Austria; Division of Cardiology, Department of Internal Medicine II, Medical University of Vienna, Vienna, Austria; Ludwig Boltzmann Institute for Lung Health, Site Penzing of Clinic Ottakring, Sanatoriumstraße 2, A-1140 Vienna, Austria; Department of Respiratory and Pulmonary Diseases, Site Penzing of Clinic Ottakring, Vienna Healthcare Group, Vienna, Austria; Center for Medical Physics and Biomedical Engineering, Medical University of Vienna, Währinger Gürtel 18-20, AKH-4L, A-1090 Vienna, Austria; Ludwig Boltzmann Institute for Cardiovascular Research, Institute of Vascular Biology and Thrombosis Research, Schwarzspanierstraße 17 H, A-1090 Vienna, Austria; Austrian Cluster for Tissue Regeneration, Ludwig Boltzmann Institute for Traumatology, Donaueschingenstraße 13, A-1200 Vienna, Austria

**Keywords:** Artificial intelligence, Electrocardiogram (ECG), AI–ECG age, Cardiovascular disease

## Abstract

**Aims:**

Artificial intelligence (AI) models trained on electrocardiograms (ECGs) can estimate age, with deviations from chronological age (Δage) serving as markers of accelerated cardiovascular ageing. We evaluated whether transformer-based AI models trained on globally diverse, heterogeneous ECG datasets outperform convolutional neural network (CNN)-based models in generalizability and physiological interpretability.

**Methods and results:**

Using 11 public datasets (435 203 recordings) from four continents and an independent external cohort (14 462 recordings), we trained and validated transformer and CNN architectures. The transformer model trained on global data achieved strong correlation with chronological age and maintained robust performance in the external cohort (*r* = 0.90), outperforming both a CNN trained on global data and a model trained exclusively on a homogenous population-based cohort (*r* = 0.88). Individuals with Δage ≥+8 years exhibited a two-fold increased mortality risk (hazard ratio = 2.08, 95% confidence interval 1.93–2.24). Explainability analysis identified the P-wave, particularly in lead V1, as the dominant contributor to age prediction, consistent with known electrophysiological changes associated with atrial remodelling and ageing. The model’s attribution to early atrial conduction features was consistent across age strata, peaking in midlife (30–50 years), and correlated positively with disease burden, including hypertension and smoking.

**Conclusion:**

Our findings establish that transformer models trained on globally heterogeneous data generalize well and can provide insights to plausible ageing signatures. Furthermore, Δage may offer insight into underlying physiological ageing processes and associated risk, though further validation is needed to define its clinical utility in risk stratification.

## Graphical Abstract

**Graphical Abstract:**
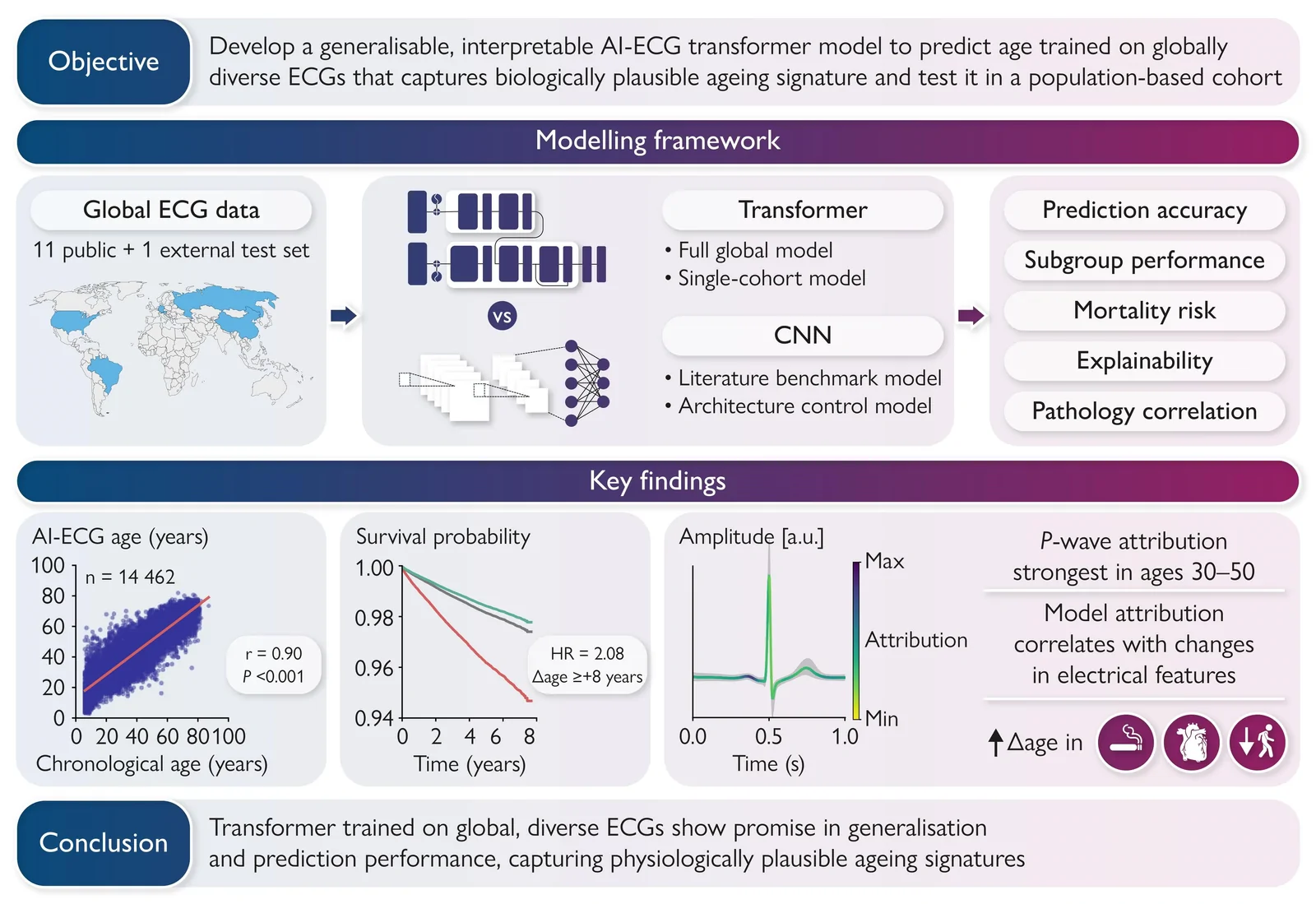

## Introduction

Artificial intelligence (AI) has become an essential tool in electrocardiogram (ECG) analysis, enabling automated feature extraction and predictive modelling.^1^ Deep neural networks have demonstrated the ability to estimate an individual’s age from ECG recordings, revealing that AI-predicted ECG age (AI-ECG age) often deviates from chronological age, a difference referred to as ‘delta age’ (Δage).^2^ Studies suggest that Δage serves as a biomarker for mortality risk, potentially capturing accelerated ageing and underlying cardiovascular health.^3^

However, the generalizability of these findings remains uncertain. Most existing models are trained on homogenous cohorts stemming from a single country or continent, limiting their applicability to global, diverse populations. This is further complicated by inter-population variability in ECG morphology (influenced by ethnicity,^4^ age distribution,^5^ and recording conditions^6^), which can degrade model performance and introduce bias. Although almost all current literature and approaches rely on convolutional neural networks (CNNs) for AI–ECG age prediction,^7^ transformers, with their global attention mechanism, may be better suited to learn complex, long-range dependencies and generalize across diverse data distributions, a key advantage over CNNs in heterogeneous, global ECG data.^8^

Yet, beyond architecture, the central question remains: Can AI–ECG age be translated into a clinically actionable biomarker? While Δage correlates with mortality risk, the underlying physiological drivers, whether lifestyle or pathological, remain poorly understood, partly due to the black-box nature of deep learning.^9^ Saliency maps and other explainability techniques have begun to identify ECG regions contributing to elevated Δage,^10,11^ yet these insights remain insufficiently mechanistic. Equally critical is understanding how lifestyle factors and comorbid cardiovascular conditions modulate AI–ECG age, a knowledge gap that constrains clinical translation.^12^

To address these challenges, the study goal was to develop a generalizable and interpretable AI model for AI-ECG age prediction that performs robustly across diverse global populations and to evaluate whether transformer architectures outperform CNNs in such heterogeneous settings. Therefore, we evaluated (1) whether a transformer-based model trained on globally diverse ECGs could generalize better than CNNs in predicting AI-ECG age across an unseen population-based cohort; (2) whether training on a single, homogeneous cohort compromises predictive performance or biological plausibility compared to models trained on heterogeneous data; (3) whether interpretability techniques reveal where transformer models capture physiologically meaningful ageing signatures, distinct from noise or pathology-specific artefacts; and whether these align with established cardiovascular ageing mechanisms. By jointly advancing generalizability and interpretability, this study seeks to bridge the gap between AI–ECG age and tangible clinical utility.

## Methods

### Datasets and pre-processing

A total of 12 datasets were utilized in this study, comprising 11 publicly available datasets^13–16^ and 1 proprietary dataset (*Table 1*). The public datasets encompass 435 203 ECGs from 320 226 subjects stemming from four different continents representing various age ranges while maintaining a balanced distribution with respect to gender. These datasets were selected because they are widely used in ECG-based machine learning studies, supporting comparability and reproducibility.^19^ Combining them brings not only diversity in population characteristics but also in recording conditions and labelling protocols, enhancing model robustness and generalizability. No subjects were excluded from the analysis due to age, deliberately to include data from all age groups and expose the model to all forms of ECGs. Prevalence of comorbidities is given in Supplementary material online, *Tables S1* and *S2*. The proprietary external population-based test set is the prospective observational registry from the LEAD (Lung, hEart, sociAl, boDy) study (ClinicalTrials.gov identifier: NCT01727518, ethics committee of Vienna Nr.: EK-11-117-0711), which contains 10-s ECGs from 14 462 individuals between 6 and 80 years collected between 2012 and 2016 as a representative sample for all age groups, genders, and socioeconomic strata in Austria.^16^ Inclusion as well as exclusion criteria of the respective datasets can be found in their respective original publications. ECGs from all datasets underwent the same pre-processing routine. They were resampled to 400 Hz using polyphase filtering, zero-padded to 4096 samples, and normalized per lead using Min–Max scaling to an amplitude between −2 and +2 to comply with the pre-processing of Lima *et al*.^3^ Recordings longer than the target input length were centre-cropped. No multiple segments per recording were used. Cardiovascular pathologies were mapped between datasets using the Systematized Nomenclature of Medicine Clinical Terms codes^20^ (see Supplementary material online, *Table S3*).

**Table 1:** Datasets characteristics

Dataset	Country	Subjects (ECGs)	Sampling Frequency [Hz]	ECG Length [s]	Mean Age (Range) [years]	Female/Male [%]
CODE-15^13^	BRA	233 770 (345 779)	400	7 or 10	53.2 (17–100)	60/40
SaMiTrop^14^	BRA	1631 (1631)	400	7 or 10	59.4 (26–98)	67/33
CPSC^17^	CHN	6868 (6868)	500	6–60	60.2 (1–92)	46/54
CPSC extra^17^	CHN	3450 (3450)	500	6–60	63.8 (1–92)	47/53
INCART^17^	RUS	32 (74)	257	1–800	56.0 (18–80)	46/54
PTB^17^	GER	290 (507)	1000	10–120	56.3 (17–87)	27/73
PTB-XL^17^	GER	18 869 (21 544)	500	10	59.5 (2–89)	48/53
Georgia^17^	USA	10 267 (10 267)	500	10	60.5 (14–89)	46/54
Ningbo^17^	CHN	34 602 (34 602)	500	10	58.1 (4–89)	43/57
Shaoxing^17^	CHN	10 247 (10 247)	500	10	60.1 (4–89)	44/56
LUDB^18^	RUS	234 (234)	500	10	52.4 (11–89)	41/59
LEAD^16^	AUT	14 462 (14 462)	500	10	38.3 (6–87)	53/47

Note: ECG, electrocardiogram; chronological age in years (mean (range)).

### Transformer model selection and training

A multivariate time series transformer was implemented as proposed by Zerveas *et al*.^21^ and optimized using five-fold cross-validation, for which all public datasets were divided into five equal-sized sets. In each iteration, three sets were used for training, one for validation, and one for testing (see Supplementary material online, *Figure S1*). To ensure that testing primarily assessed generalization on external datasets, two considerations were made during dataset partitioning: First, individual datasets were not split across sets, except the CODE-15 dataset necessitating division for balanced sampling due to its size and second, datasets with similar origin were assigned to the same set. For datasets encompassing multiple ECGs per subject, a subject-level split was performed such that ECGs of the same subject would only either be in train, validation, or test sets to avoid data leakage. Repeated ECGs of the same subjects were held together within the same set. Hyperparameters were optimized via random search followed by a tree-structured Parzen estimator.^22^ Exact set of tested hyperparameters can be found in Supplementary material online, *Table S4*. The best combination used ReLU activations, batch size 64, and RAdam optimizer.^23^ The initial transformer layer employed a 1D CNN with optimized kernel size, padding, and stride. Batch normalization followed convolution and was applied within encoder layers, with dropout after positional encoding and attention. Training used up to 128 epochs with early stopping after 10 epochs of no validation loss improvement to avoid overfitting (see Supplementary material online, *Table S5*). For all models, the root mean squared error between the chronological age and AI–ECG age was used as training and validation loss metric. The best model was determined based on the mean absolute error (MAE) between AI–ECG age and chronological age.

### Evaluation

We compared AI-ECG age obtained from four different modelling approaches (*Figure 1*): 1) The multivariate time series transformer model trained using five-fold cross-validation and hyperparameter optimization on all public datasets, referred to as the transformer trained on global datasets (Transformer (Global)). 2) To investigate the model’s performance for a single, homogenous population-based cohort the same model was trained on the population-based LEAD dataset (Transformer (LEAD)). 3) As a benchmark, a pre-trained CNN model from the literature developed by Lima *et al*.^3^ was used, since it is currently regarded as the state-of-the-art model within this field of research and was being used in the majority of publications in recent years.^7^ The model was tested on the public datasets and was referred to as CNN (Lima). 4) To isolate the effects attributable to datasets composition vs. model architecture, a CNN model was trained on all public datasets using the same procedure as Lima *et al*.^3^ and was tested on all public datasets, referred to as CNN trained on global datasets (CNN (Global)). Model performance was assessed on the test sets of the public datasets that were left out of training and validation during the five-fold cross-validation approach as well as on the LEAD dataset to show their capability to predict AI–ECG age in an external population-based cohort. To separate training volume from dataset heterogeneity, we trained a size-matched variant of the Transformer (Global), subsampling the global training set to the LEAD cohort’s size while holding architecture, hyperparameters, five-fold cross-validation, and the proportions of all eleven datasets constant. The global vs. size-matched gap reflects volume; the size-matched vs. LEAD gap, heterogeneity.

**Figure 1:**
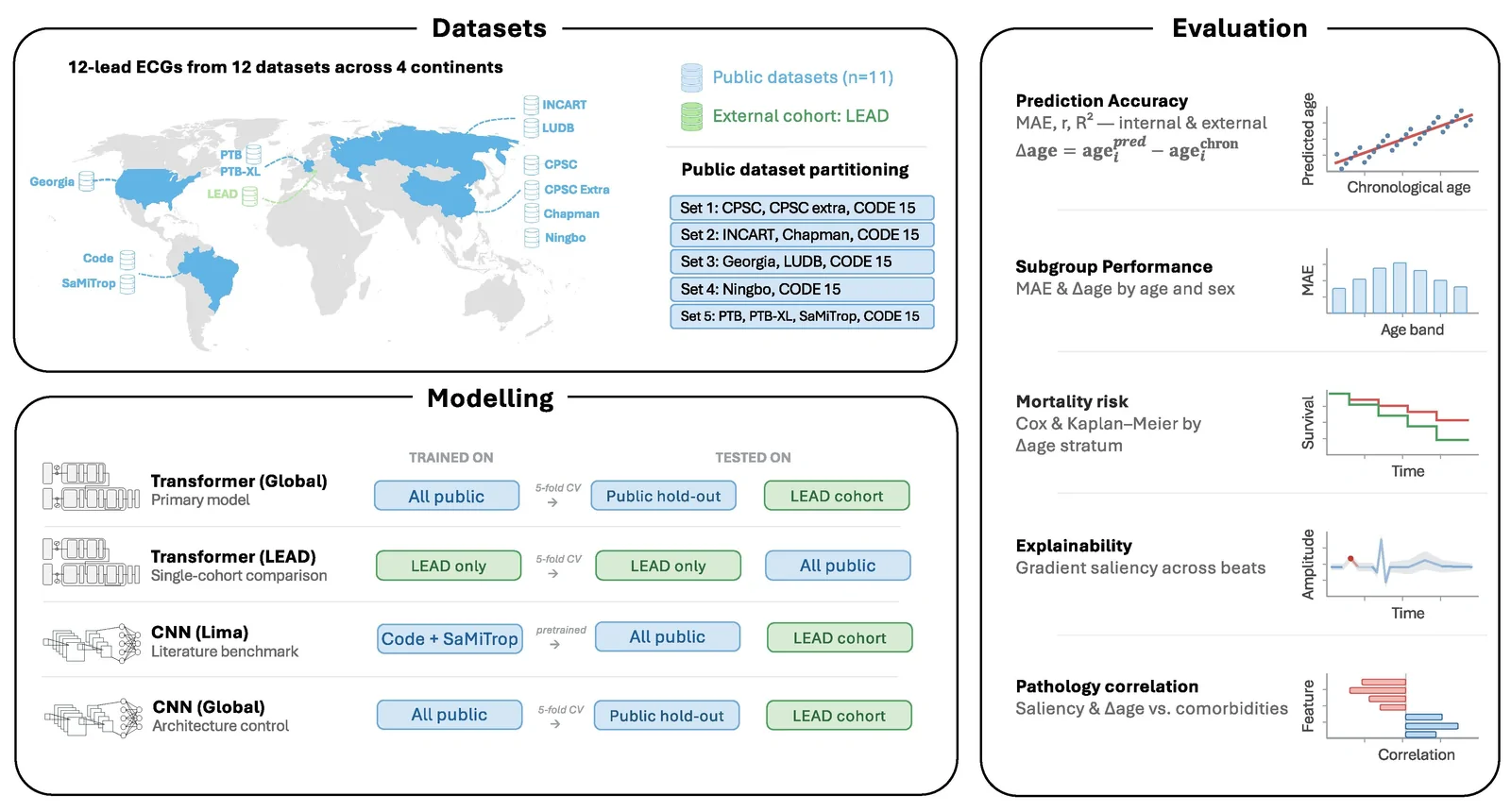
*Study overview. Datasets:* 12-lead electrocardiograms from 12 datasets across four, from which were 11 public (blue) for model development and the external population-based LEAD cohort (green). *Modelling:* the four compared models (Transformer and convolutional neural network, each trained on all-public or single-cohort data), with the data each was trained and tested on, coloured by origin. *Evaluation:* prediction accuracy, subgroup performance, mortality risk, saliency-based explainability, and pathology correlation.

Survival analyses were performed in CODE-15 and SaMiTrop, the only two datasets with longitudinal mortality data. Subjects were first stratified by Δage into accelerated (Δage ≥+8 years), concordant (−8 to +8 years) and decelerated (Δage ≤−8 years) ageing, using the 8-year threshold of Lima *et al*.^3^ for comparability. Overall survival was compared with Kaplan–Meier estimates and log-rank tests, with stratum-specific hazard ratios (HRs) from Cox proportional-hazards models adjusted for chronological age and sex. To quantify the continuous, independent and incremental prognostic value of Δage, and to test whether it is robust to measured cardiac disease and consistent across model architectures, nested Cox models were fitted in both cohorts for the Transformer (Global) and CNN (Lima) in four adjustment levels: chronological age and sex (M1); + standardized Δage (M2); + a normal-ECG flag (M3); and + binary ECG diagnoses (M4). The primary estimate was HR per 1-SD increase in Δage. Discrimination was assessed using Harrell’s concordance index (C-index) with bootstrapped 95% CIs (100 resamples). The incremental value of Δage over the age-and-sex baseline (M1 vs. M2) was quantified by a likelihood-ratio test and the change in C-index (ΔC-index). Finally, the two architectures were compared using a paired bootstrap on identical resamples, yielding a paired ΔC-index with a 95% confidence interval (CI) and a two-sided *P*-value.

To evaluate and compare the predictive performance of the AI–ECG models, pairwise differences in predicted cardiac age were analysed for each dataset. Normality of these paired differences was assessed using the D’Agostino–Pearson test. Statistical significance was determined using either a paired *t*-test (for normally distributed differences) or a Wilcoxon signed-rank test (for non-normal distributions). Bonferroni correction was applied to adjust for multiple comparisons. The relationship between chronological age and AI–ECG age was assessed using Pearson correlation coefficients, corresponding *P*-values, and coefficients of determination, calculated separately for each model and dataset. Subgroup analyses were conducted to examine demographic biases and performance variability across pooled and individual datasets. Populations were stratified by gender and into 10-year age intervals (0–100 years). For each subgroup, Δage and MAE were computed and reported as means with SDs and 95% CIs. Additionally, subgroup-specific Pearson correlations with 95% CIs, derived using Fisher’s *z*-transformation, were calculated to assess consistency across age and gender groups.

To interpret the model’s predictions and identify temporally critical ECG segments, saliency maps were computed using the vanilla gradient method^24^—established as a reliable explainable AI approach for ECG-based classification.^10^ Saliency maps were derived using the Captum library for PyTorch,^25^ with gradients explicitly enabled for backpropagation from output to input time series. Raw input gradients were directly extracted for the target class, without smoothing or noise injection.

Saliency values were aggregated across heartbeats and individuals, with subjects grouped by chronological age and attributions averaged per heartbeat. ECGs were delineated using Neurokit2^26^ using a discrete wavelet transform algorithm^27^ to segment P-wave, PQ segment, QRS complex, ST segment, and T-wave. Beats were aligned to R-peaks, and individual cardiac cycles were reconstructed around each, with P-, QRS-, and T-wave boundaries assigned within physiologically plausible, R–R-interval-scaled search windows (details in Supplementary material). Morphological and global rhythm features were extracted per segment and lead. Beats with abnormal interval or amplitude were discarded; recordings where delineation failed were excluded from explainability analysis. To ensure no systematic bias, included and excluded recordings were compared across all datasets by age, sex, origin, rhythm, and major ECG findings.

To assess whether model attributions or age prediction gaps reflect specific cardiovascular or systemic pathologies, subgroup analyses were conducted within the LEAD cohort. Participants were stratified by lifestyle factors and comorbidities. A healthy reference group (absence of comorbidities and presence of healthy lifestyle) served as baseline. Physiological drivers of attributions were evaluated via Spearman’s rank correlation between localized saliency and extracted ECG features, identifying pathology-specific morphological influences. Model predictions were further analysed using two-sided Mann–Whitney *U* tests to compare AI–ECG age and Δage across disease subgroups vs. the healthy reference, accommodating non-normal variances. Subgroup-specific correlations between chronological and AI–ECG age (Spearman’s *ρ* and MAE) quantified pathology-driven acceleration of predicted AI–ECG age. To control for demographic confounders such as differences in age between subpopulations (Supplementary material online, *Figure S3*), subpopulations were compared against an age- and sex-matched healthy control group.

## Results

### Model performance and external validation

Our analysis demonstrates that transformer-based architectures trained on globally diverse ECG cohorts (Transformer (Global)) achieve performance comparable to state-of-the-art pretrained CNNs (CNN (Lima)) in predicting AI–ECG age across multiple public datasets, with slightly lower MAEs in most cases (*Table 2*). Notably, the Transformer (Global) model achieved the lowest MAE in 6 of the 12 datasets, underscoring its robustness when trained on heterogeneous, global data. In contrast, the model trained on a single, homogenous cohort exhibited higher MAEs across nearly all datasets, particularly in smaller or more clinically distinct cohorts such as INCART (MAE = 81.5 years) in comparison to the Transformer (Global) (MAE = 14.3 years), indicating significant performance degradation when trained on limited, population-specific data. A consistent trend across models was systematic underestimation of AI-ECG age in older individuals, reflected in negative mean Δage values (*Table 2*). This bias was most pronounced in the Transformer (LEAD) model (Δage = −5.4 years in INCART) and CNN (Lima) model (Δage = 2.3 years in INCART), though the Transformer (Global) model also exhibited negative Δage (−0.9 to −2.5 years) in several cohorts, suggesting that even globally trained models may inherit age-related systematic drift (see Supplementary material online, *Figure S4*). Skewed age distribution across datasets and cross-validation splits towards the fifth to seventh decades, with sparse representation of patients under 30, though this shape held across all folds (see Supplementary material online, *Figures S12* and *S13*), may explain this regression-to-the-mean pattern.

**Table 2:** Model performances in terms Δage, MAE and correlation coefficients across datasets

Parameter	Dataset	Model			
		Transformer (Global)	Transformer (LEAD)	CNN (Lima)	CNN (Global)
Δage	CODE-15	−0.9 (11.0) [−1.0 to 0.9]	6.0 (24.8) [5.9–6.0]	0.9 (11.1) [0.8–0.9]	−0.4 (16.8) [−0.5 to 0.4]
	SaMiTrop	2.4 (11.9) [1.8–3.0]	4.7 (23.6) [3.5–5.8]	3.2 (12.1) [2.6–3.8]	−2.8 (14.0) [−3.5 to 2.1]
	LUDB	−0.4 (11.5) [−1.9 to 1.1]	1.5 (17.6) [−0.8 to 3.7]	0.9 (12.1) [−0.7 to 2.4]	−2.0 (16.0) [−4.0 to 0.1]
	Shaoxing	−1.2 (11.1) [−1.4 to 0.9]	−10.4 (16.6) [−10.7 to 10.1]	1.4 (14.2) [1.1–1.6]	−11.2 (18.9) [−11.6 to −10.9]
	CPSC	−1.5 (10.8) [−1.8 to 1.3]	−5.9 (16.2) [−6.3 to 5.5]	3.9 (12.4) [3.6–4.2]	4.4 (18.6) [4.0–4.9]
	CPSC extra	−2.5 (11.2) [−2.9 to 2.1]	−5.2 (15.9) [−5.8 to 4.7]	3.8 (12.6) [3.4–4.2]	6.5 (18.3) [5.9–7.1]
	Georgia	−1.3 (13.1) [−1.6 to 1.1]	−7.1 (16.9) [−7.4 to 6.8]	3.4 (14.3) [3.2–3.7]	−2.9 (20.0) [−3.3 to 2.5]
	Ningbo	0.6 (11.9) [0.5–0.7]	−8.7 (17.9) [−8.9 to 8.5]	2.6 (15.7) [2.4–2.7]	−0.2 (12.7) [−0.3 to −0.0]
	PTB-XL	−0.7 (10.6) [−0.8 to 0.6]	−6.5 (15.4) [−6.7 to 6.3]	2.0 (11.3) [1.9–2.2]	−3.5 (12.8) [−3.7 to 3.4]
	PTB	−0.9 (10.7) [−1.8 to 0.0]	−5.1 (16.0) [−6.5 to 3.8]	5.2 (11.7) [4.2–6.2]	−6.5 (12.6) [−7.6 to 5.4]
	INCART	−5.4 (17.9) [−9.5 to 1.3]	77.9 (104.2) [54.2–101.7]	2.3 (16.7) [−1.5 to 6.1]	−10.1 (30.7) [−17.1 to 3.1]
	LEAD	4.0 (9.2) [3.9–4.2]	−0.7 (10.0) [−0.9 to −0.6]	5.7 (11.1) [5.5–5.9]	−0.3 (11.6) [−0.5 to −0.1]
MAE	CODE-15	8.5 (7.1) [8.5–8.5]	17.5 (18.5) [17.5–17.6]	8.5 (7.2) [8.5–8.5]	13.2 (10.5) [13.1–13.2]
	SaMiTrop	9.4 (7.6) [9.0–9.8]	17.4 (16.6) [16.6–18.2]	10.0 (7.6) [9.6–10.3]	11.2 (8.9) [10.8–11.6]
	LUDB	9.2 (6.8) [8.4–10.1]	12.4 (12.5) [10.8–14.0]	9.7 (7.3) [8.7–10.6]	12.8 (9.7) [11.6–14.1]
	Shaoxing	8.4 (7.4) [8.3–8.6]	15.2 (12.4) [14.9–15.4]	10.7 (9.4) [10.6–10.9]	18.0 (12.5) [17.8–18.3]
	CPSC	8.5 (6.9) [8.3–8.6]	13.1 (11.1) [12.9–13.4]	10.0 (8.2) [9.9–10.2]	15.0 (11.9) [14.7–15.3]
	CPSC extra	8.8 (7.3) [8.6–9.1]	12.6 (11.0) [12.3–13.0]	10.0 (8.5) [9.7–10.3]	15.4 (11.9) [15.0–15.8]
	Georgia	10.3 (8.2) [10.1–10.4]	14.1 (11.6) [13.9–14.4]	11.4 (9.3) [11.2–11.6]	16.1 (12.1) [15.9–16.4]
	Ningbo	9.0 (7.9) [8.9–9.1]	15.2 (12.9) [15.0–15.3]	11.3 (11.2) [11.2–11.4]	9.7 (8.2) [9.7–9.8]
	PTB-XL	8.3 (6.6) [8.2–8.4]	12.5 (11.1) [12.3–12.6]	9.0 (7.2) [8.9–9.1]	10.4 (8.3) [10.3–10.5]
	PTB	8.4 (6.7) [7.9–9.0]	13.0 (10.7) [12.0–13.9]	10.2 (7.8) [9.5–10.9]	11.3 (8.6) [10.5–12.0]
	INCART	14.3 (12.0) [11.6–17.1]	81.5 (101.4) [58.4–104.6]	14.0 (9.3) [11.9–16.1]	21.8 (23.7) [16.4–27.2]
	LEAD	7.9 (6.2) [7.8–8.0]	7.5 (6.6) [7.4–7.6]	10.1 (7.4) [10.0–10.2]	9.4 (6.8) [9.3–9.5]
*r, P-value, r^2^*	CODE-15	0.83, <0.001, 0.69	0.33, <0.001, 0.11	0.59, <0.001, 0.35	0.59, <0.001, 0.35
	SaMiTrop	0.56, <0.001, 0.32	0.08, 0.001, 0.01	0.6, <0.001, 0.36	0.31, <0.001, 0.09
	LUDB	0.8, <0.001, 0.65	0.58, <0.001, 0.33	0.8, <0.001, 0.63	0.6, <0.001, 0.36
	Shaoxing	0.77, <0.001, 0.59	0.54, <0.001, 0.29	0.69, <0.001, 0.48	0.66, <0.001, 0.44
	CPSC	0.82, <0.001, 0.68	0.62, <0.001, 0.38	0.8, <0.001, 0.63	0.61, <0.001, 0.38
	CPSC extra	0.7, <0.001, 0.48	0.45, <0.001, 0.2	0.67, <0.001, 0.45	0.44, <0.001, 0.2
	Georgia	0.59, <0.001, 0.34	0.46, <0.001, 0.21	0.6, <0.001, 0.36	0.38, <0.001, 0.14
	Ningbo	0.8, <0.001, 0.64	0.58, <0.001, 0.33	0.68, <0.001, 0.46	0.77, <0.001, 0.6
	PTB-XL	0.77, <0.001, 0.6	0.6, <0.001, 0.36	0.78, <0.001, 0.61	0.65, <0.001, 0.42
	PTB	0.72, <0.001, 0.53	0.41, <0.001, 0.17	0.71, <0.001, 0.5	0.51, <0.001, 0.26
	INCART	0.39, 0.001, 0.15	−0.16, 0.172, 0.03	0.49, <0.001, 0.24	0.27, 0.021, 0.07
	LEAD	0.9, <0.001, 0.81	0.88, <0.001, 0.78	0.85, <0.001, 0.72	0.84, <0.001, 0.71

For each model (Transformer (Global), Transformer (LEAD), CNN (Lima), and CNN (Global)), Δage (mean (SD) [95% CI]) represents the difference between predicted age and chronological age, while MAE (mean (SD) [95% CI]) quantifies the average absolute deviation between predicted and actual age.

Notably, the Transformer (LEAD) model displayed the greatest variance in prediction range (e.g. Δage 95% CI −0.8 to +3.7 years in LUDB, and 54.2 to 101.7 years in INCART), suggesting reduced stability and generalizability compared to the Transformer (Global) or the CNN (Lima) model. The CNN (Lima) model outperformed the CNN (Global) model in only three datasets, while the CNN (Global) model outperformed the CNN (Lima) in four datasets, suggesting that fine-tuning on public datasets can be beneficial and that the choice of architecture and training strategy must be context dependent. The Transformer (Global) model showed the strongest correlation with chronological age in the LEAD dataset (*r* = 0.90, *P* < 0.001), outperforming the CNN (Global) (*r* = 0.83, *P* < 0.001) and the Transformer (LEAD) model (*r* = 0.88, *P* < 0.001). For correlations of all four models for all datasets, see Supplementary material online, *Figure S5*.

The size-matched model outperformed the Transformer (LEAD) on pooled datasets, showing heterogeneity improves generalization independently of volume. Matching training size of the Transformer (LEAD) raised MAE from 8.6 to 11.5 years. Both factors thus contribute, with heterogeneity providing larger share of the advantage over the cohort-specific model on diverse public populations. On the LEAD cohort itself, the Transformer (LEAD) was best (7.5 years) and the size-matched model worst (13.2 years); only the full-volume Transformer (Global) approached cohort-specific performance (7.9 years), underscoring that its generalization to a single narrow cohort depends on large training volume (*Table 3*).

**Table 3:** Isolating training volume from dataset heterogeneity in transformer-based AI-ECG age prediction

Dataset	MAE_size-matched_	ΔMAE_volume_	p-value	ΔMAE_heterogeneity_	*P*-value
CODE-15	11.6 (9.0) [11.6–11.7]	3.1 (8.5) [3.1–3.2]	<0.001	−5.9 (18.4) [−5.9 to −5.8]	<0.001
SaMiTrop	10.9 (8.5) [10.5–11.3]	1.5 (8.5) [1.1–1.9]	<0.001	−6.5 (17.0) [−7.3 to −5.7]	<0.001
LUDB	10.4 (7.8) [9.3–11.5]	1.1 (6.3) [0.3–2.0]	0.09	0.4 (8.3) [−0.7–1.6]	0.49
Shaoxing	10.7 (8.4) [10.6–10.9]	2.3 (8.4) [2.1–2.5]	<0.001	−4.4 (12.1) [−4.7 to −4.2]	<0.001
CPSC	11.2 (8.8) [10.9–11.4]	2.7 (8.3) [2.5–2.9]	<0.001	−2.0 (10.2) [−2.2 to −1.8]	<0.001
CPSC extra	10.8 (8.8) [10.5–11.1]	2.0 (8.1) [1.8–2.3]	<0.001	−1.8 (9.8) [−2.1 to −1.5]	<0.001
Georgia	12.4 (9.6) [12.2–12.6]	2.1 (9.1) [1.9–2.3]	<0.001	−1.8 (10.6) [−2.0 to −1.6]	<0.001
Ningbo	11.5 (9.4) [11.4–11.6]	2.5 (7.9) [2.4–2.6]	<0.001	−3.7 (13.0) [−3.8 to −3.5]	<0.001
PTB-XL	10.4 (8.1) [10.3–10.5]	2.1 (7.4) [2.0–2.2]	<0.001	−2.1 (10.7) [−2.2 to −1.9]	<0.001
PTB	10.2 (7.6) [9.5–10.9]	1.8 (8.1) [1.1–2.5]	<0.001	−2.8 (11.1) [−3.7 to −1.8]	<0.001
INCART	17.6 (12.0) [14.8–20.3]	3.2 (16.0) [−0.4 to 6.9]	0.09	−63.9 (105.6) [−88.0 to −39.9]	<0.001
LEAD	13.2 (9.9) [13.0–13.4]	5.3 (8.1) [5.1–5.4]	<0.001	5.7 (11.0) [5.5–5.9]	<0.001
Pooled	11.5 (9.0) [11.5–11.6]	3.0 (8.4) [2.9–3.0]	<0.001	−5.3 (17.4) [−5.3 to −5.2]	<0.001

Mean absolute error (MAE, in years; reported as mean (SD) [95% CI]) of Transformer (Global) model retrained on a random subsample of the global training pool matched to the LEAD training size (MAE_size-matched_). ΔMAE_volume_ = MAE_size-matched_ − MAE_Global_ isolates the accuracy lost by reducing training volume alone (positive values indicate degradation). ΔMAE_heterogeneity_ = MAE_size-matched_ − MAE_LEAD_ compares heterogeneous vs. cohort-specific training at equal volume (negative values favour the heterogeneous model).

Analysis of the pooled subgroup model performance stratified by chronological age and sex showed that the Transformer (Global) outperformed the model trained on the LEAD cohort across all age strata (*Figure 2*). Furthermore, it demonstrated significantly lower MAEs, ranging from 6.89 to 20.16, compared to Transformer (LEAD) model’s 11.90 to 24.75, with the largest disparities observed in adolescents (10–20 years, MAE = 7.30 vs. 18.86) and the elderly (80–90 years, 11.94 vs. 20.97). Δage estimates were also more aligned with chronological age in the Transformer (Global) model: e.g. in 30–50-year-olds, a Δage between 3.26 and 5.13 was observed, while the Transformer (LEAD) model overestimated by 8.19–16.83, suggesting a bias towards older phenotypes. Correlation analyses revealed that the Transformer (Global) model consistently showed stronger associations (*r* = 0.21–0.24 in 20–60 years) than the Transformer (LEAD) model (*r* = 0.04–0.19). Notably, the Transformer (Global) model showed no correlation (*r* = 0.01) in 10–20-year-olds, potentially reflecting the absence of age-specific ECG signatures in early adulthood and the limited density of young adult samples in the pooled training distribution.

**Figure 2:**
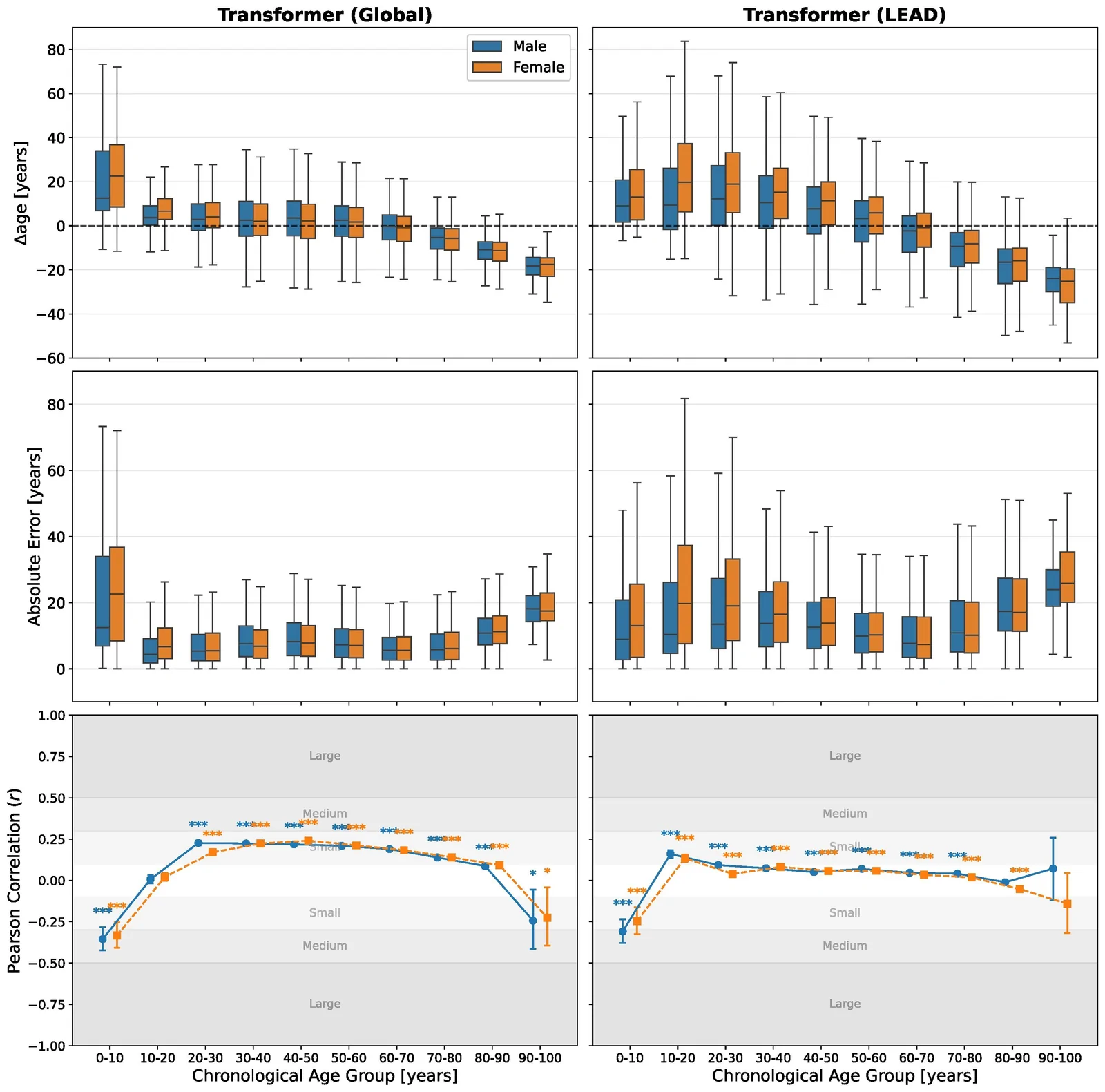
Pooled subgroup analysis of model performance stratified by chronological age and biological sex. The analysis compares the two transformer models’ (Transformer (Global), left, Transformer (LEAD), right) performance averaged across datasets for male (blue) and female (orange). (Top): Δage, defined as artificial intelligence–electrocardiogram age minus chronological age, across chronological age deciles. (Middle): Distribution of mean absolute error (MAE) across age deciles. (Bottom): Pearson correlation coefficients (*r*) between predicted and chronological age within each gender across age deciles. Error bars represent 95% confidence intervals and statistical significance indicated as *<0.05, **<0.01, and ***<0.001. Shaded grey background regions correspond to Cohen’s standard effect size thresholds for correlation magnitude (Small: ∣r∣=0.1–0.3, Medium: ∣r∣=0.3–0.5, Large: ∣r∣>0.5).

### Survival analysis

On the CODE-15 dataset (*n*_events_ = 8344, mean follow-up: 3.7 years), individuals with Δage ≥+8 years exhibited a larger HR (2.08 [1.93–2.24 95% CI], *P* < 0.001) compared to those with Δage ≤−8 (HR = 0.85, [0.81–0.90 95% CI], *P* < 0.001) (*Figure 3*) (pairwise log-rank *P* < 0.001, Supplementary material online, *Table S6*). Those with Δage between −8 and +8 years served as the reference. In SaMiTrop (*n*_events_ = 104, mean follow-up: 3.4 years), the HR was elevated for Δage ≥+8 years (HR = 2.07, [1.24–3.45 95% CI], *P* = 0.005). Notably, Δage ≤−8 years showed no significant increased HR in either cohort (SaMiTrop HR = 1.25, *P* = 0.377). Furthermore, the difference between the two age categories was just not statistically significant (pairwise log-rank *P* = 0.062).

**Figure 3:**
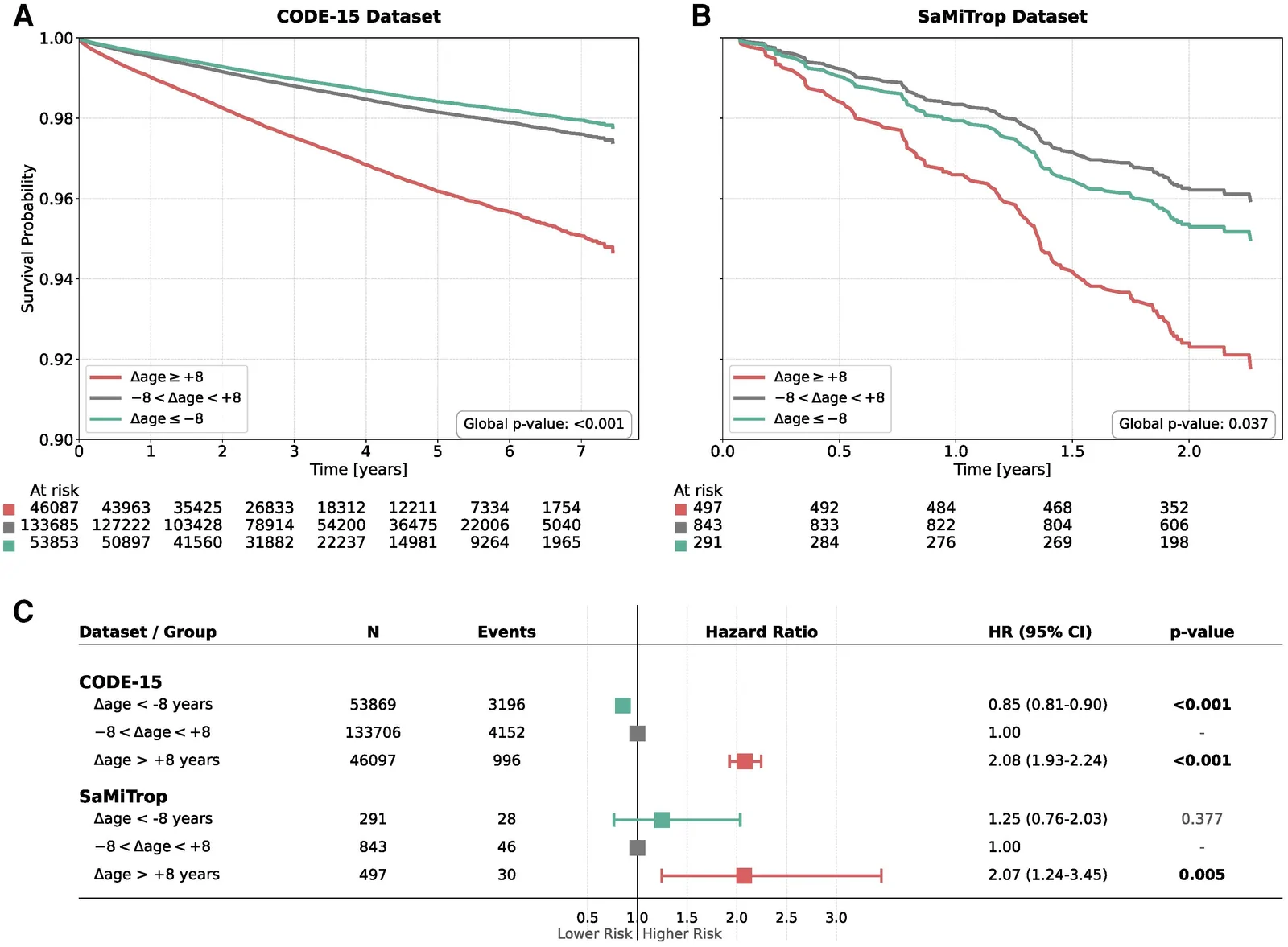
Survival analysis and mortality risk stratification based on Δage. (*A*) Kaplan–Meier overall survival estimates for the CODE and (*B*) SaMiTrop datasets. Patients are stratified into three distinct groups based on Δage: decelerated ageing (Δage ≤−8 years; green), concordant age (−8 years < Δage < +8 years; grey) and accelerated ageing (Δage ≥+8 years; red). The number of patients at risk over time (in years) is populated below each axis. Global statistical variance among the strata was determined using a multivariate log-rank test. (*C*) Forest plot depicting the associated relative risk of all-cause mortality derived from Cox proportional hazards regression models utilizing the transformer model predictions across both cohorts. The concordant aging group (−8 years<Δage <+8 years) serves as the baseline reference presenting standard risk. HR, hazard ratio; CI, confidence interval.

When evaluated as a continuous metric, Δage remained a strong independent predictor of mortality (*Table 4*). For every 1-SD increase in Δage (1 SD = 11.0 years CODE-15; 11.9 years SaMiTrop) the risk of death increased significantly (CODE-15: HR = 1.30 [1.26–1.33 95%CI], *P* < 0.001; SaMiTrop: HR = 1.38 [1.07–1.77 95% CI], *P* = 0.011). Furthermore, the addition of Δage to a baseline model of chronological age and sex improved prognostic accuracy in the CODE-15 dataset (ΔC-index = +0.004, likelihood ratio <0.001). Across Cox models with covariate adjustment (*Table 4*), Δage remained an independent predictor of mortality in CODE-15. The Transformer (Global) per-SD HR attenuated only modestly after adjustment for a normal-ECG flag and ECG diagnoses (from 1.30 to 1.25; all *P* < 0.001), indicating prognostic information beyond overt ECG disease. In comparison to the Transformer discrimination for the CNN (Lima) was marginally above, and statistically indistinguishable (paired ΔC-index = −0.004; C-index = 0.799 vs. 0.803 at M2). In SaMiTrop, for the Transformer (Global) HR was not significant after adjustment (1.27; *P* = 0.058), the architectures did not differ significantly.

**Table 4:** Nested Cox proportional hazards models evaluating the independent prognostic value of ECG-derived age gap (Δage) with progressive covariate adjustment—CODE-15 and SaMiTrop cohorts

Dataset	Model level	Architecture	HR Δage (95% CI)	*P*-value	C-index (95% CI)	LR *P* vs. M1	ΔC-index vs. M1	ΔC-index vs. CNN (95%CI)	*P* vs. CNN
Code-15	M1: Age + Sex	Transformer (Global)	—	—	0.795	—	—	—	—
		CNN (Lima)	—	—	0.795	—	—	—	—
	M2: + Δage	Transformer (Global)	1.30 (1.26–1.33)	<0.001	0.799 (0.795–0.803)	<0.001	+0.004	−0.004 (−0.005 to −0.003)	<0.001
		CNN (Lima)	1.37 (1.34–1.41)	<0.001	0.803 (0.799–0.807)	<0.001	+0.008	—	—
	M3: + Normal ECG	Transformer (Global)	1.28 (1.24–1.32)	<0.001	0.801 (0.797–0.805)	—	+0.006	−0.004 (−0.005 to −0.003)	<0.001
		CNN (Lima)	1.36 (1.33–1.39)	<0.001	0.805 (0.801–0.808)	—	+0.010	—	—
	M4: + ECG conditions	Transformer (Global)	1.25 (1.22–1.29)	<0.001	0.807 (0.803–0.811)	—	+0.012	−0.003 (−0.004 to −0.003)	<0.001
		CNN (Lima)	1.33 (1.29–1.36)	<0.001	0.810 (0.807–0.814)	—	+0.015	—	—
SaMiTrop	M1: Age + Sex	Transformer (Global)	—	—	0.684	—	—	—	—
		CNN (Lima)	—	—	0.684	—	—	—	—
	M2: + Δage	Transformer (Global)	1.38 (1.07–1.77)	<0.05	0.703 (0.668–0.760)	<0.01	+0.019	−0.012 (−0.037 to +0.003)	0.160
		CNN (Lima)	1.55 (1.24–1.92)	<0.001	0.715 (0.679–0.769)	<0.001	+0.031	—	—
	M3: + Normal ECG	Transformer (Global)	1.27 (0.99–1.64)	0.058	0.720 (0.687–0.774)	—	+0.036	−0.010 (−0.030 to +0.003)	0.140
		CNN (Lima)	1.46 (1.17–1.82)	<0.001	0.730 (0.699–0.782)	—	+0.046	—	—

Δage, standardised per 1 SD = 11.0 years in CODE-15 cohort; 11.9 years in SaMiTrop cohort; M1: age + sex (reference, no Δage); M2: + Δage; M3: + normal ECG flag; M4: + ECG rhythm diagnoses (1dAVb, RBBB, LBBB, SB, ST, AF); M4 was estimated in CODE-15 only (covariates unavailable in SaMiTrop). C-index confidence intervals were derived from 100 bootstrap resamples of the partial hazard vector. LR p vs. M1 reports the likelihood ratio test for Δage alone (M2 vs. M1, 1 df). ΔC-index vs. M1 reflects the cumulative gain in discrimination over the age-and-sex baseline for the same architecture and cohort. ΔC-index (CNN) and p (CNN) reflect a paired bootstrap comparison between the Transformer (Global) and CNN (Lima) evaluated on identical resamples; values shown on Transformer rows only.

Calibration analysis across age deciles revealed a systematic regression-to-the-mean bias, wherein the model tended to overestimate the age of younger demographics and underestimate the age of older demographics (see Supplementary material online, *Figure S6*). Overall, chronological age accounted for approximately 21% of the variance in the Δage metric (*r*^2^ = 0.209, *β* = −0.254). To ensure that the prognostic value of Δage was not an artefact of this age-dependent bias, we performed a secondary continuous Cox proportional hazards analysis using an age-residualized Δage mathematically isolating the physiological deviation from chronological age. Afterwards, the standardized Δage remained highly significant, independent predictor of all-cause mortality. In the CODE-15 dataset, each 1-SD increase in the age-residualized Δage was associated with a 26% increased HR of 1.26, 95% CI: 1.23–1.29; *P* < 0.001, achieving a C-index = 0.799. Importantly, this independent prognostic strength was confirmed in the SaMiTrop cohort (HR = 1.33, [1.07–1.66 95% CI]; *P* = 0.011; C-index = 0.703) (*Table 5*).

**Table 5:** Age-residualised continuous Cox proportional hazards analysis for mortality prediction

Dataset	Patients	Events	Β (Chronological age)	R^2^ (Age vs. Δage)	Residualised HR (95% CI)	*P*-value (Cox)	C-index
Code-15	233 672	8344	−0.254	0.209	1.26 (1.23–1.29)	<0.001	0.799
SaMiTrop	1631	104	−0.254	0.209	1.33 (1.07–1.66)	0.011	0.703

Hazard ratios (HRs) represent the change in all-cause mortality risk per 1-SD increase in age-residualised delta-age. Δage was residualised for chronological age using linear regression. C-index indicates model concordance.

### Model explainability

17.6% ECGs were excluded from the explainability analysis due to failed delineation or derived parameters outside physiologically plausible boundaries (see Supplementary material online, *Tables S7* and *S8*; *Figure S11*). Excluded ECGs were modestly older (mean 56.8 vs. 53.4; Cohen’s *d* = 0.17), more often male (51.4% vs. 39.7%), and enriched for rhythm and conduction abnormalities with correspondingly less normal sinus rhythm. However, all diagnostic categories remained well represented, any residual bias to the explainability findings was considered limited.

In the pooled analysis across all 12 ECG leads, the highest mean saliency scores were consistently observed in the P-wave and PQ-segment (*Figure 4A*; 0.127 and 0.133, respectively) (*Figure 4B*). The QRS complex (0.081) and ST segment (0.070) followed, while the T-wave exhibited the lowest saliency (0.063). These findings indicate that the highest attributions localise to early atrial and atrioventricular conduction events. Lead-specific analysis revealed that lead I and V1 exhibited the highest mean saliency across most segments (*Figure 4C*). Notably, V1 for the P-wave (0.230) and PQ segment (0.210), potentially reflecting its sensitivity to early atrial activation and interatrial conduction. Saliency scores were further stratified by chronological age decile (0–100 years). A clear age-dependent pattern emerged: saliency for all segments increased with age, peaking between 30 and 50 years, after which it gradually declined (*Figure 4C*). The most pronounced age-related increase was observed for the P-wave (from 0.120 to 0.230) and PQ segment (from 0.095 to 0.210), suggesting that the model’s attention to atrial and atrioventricular nodal conduction intensifies during midlife. While minor sex-based differences in saliency were observed (see Supplementary material online, *Figure S8*), they were generally negligible in magnitude.

**Figure 4:**
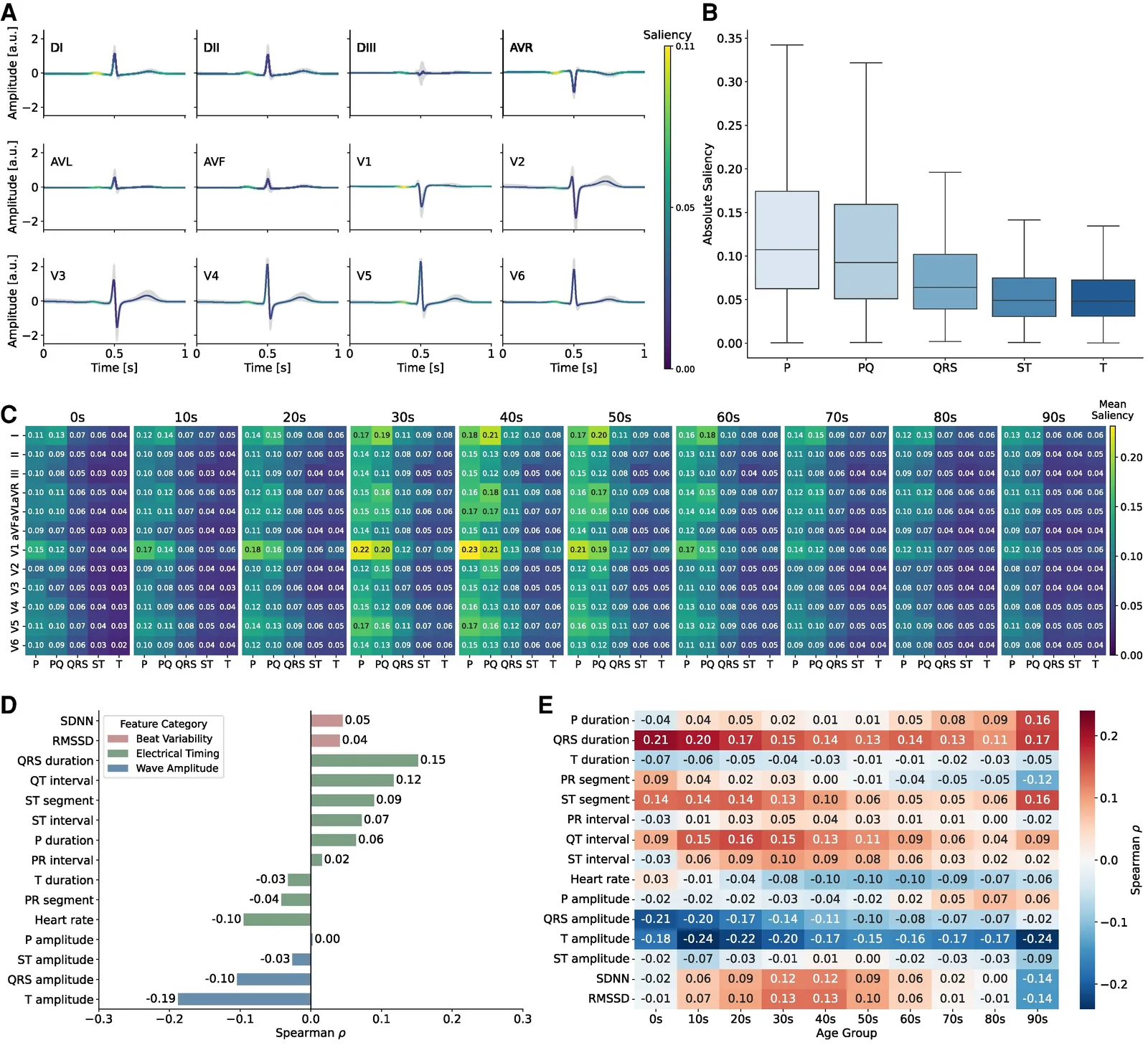
Saliency-based explainability and physiological feature sensitivity of the model. (*A*) Representative global median electrocardiogram traces for all 12 leads. The colour gradient indicates the median absolute saliency, highlighting regions of highest model focus. (*B*) Box plots showing the pooled sensitivity of the model to standardized electrocardiogram segments. (*C*) Heatmaps displaying the mean absolute saliency distributed across all leads and electrocardiogram segments, grouped by age deciles to show developmental changes in model attribution. (*D*) Global Spearman rank correlations isolating the relationship between model saliency and clinically relevant electrocardiogram features. Features are grouped by electrical timing, wave amplitude, and beat variability. (*E*) Heatmap of age-stratified Spearman correlations of electrocardiogram features and saliency.

Results of the correlation between saliency and ECG features, pooled across all datasets and leads (*Figure 4D*) revealed a positive correlation between saliency and QRS duration (*r* = 0.152, *P* < 0.001), ST segment duration (*r* = 0.089), and QT interval duration (*r* = 0.117). Conversely, saliency was negatively correlated with T-wave amplitude (*r* = −0.188, *P* < 0.001) and QRS amplitude (*r* = −0.104). Notably, saliency showed no significant correlation with heart rate or heart rate variability (HRV) parameters (SDNN, RMSSD; *r* = 0.045, 0.041, respectively).

Age-stratified correlation analyses revealed that in midlife (30–50 years), saliency for QRS duration reached its peak (*r* = 0.137–0.158), while T-wave amplitude showed a negative correlation (*r* = −0.166 to −0.237) (*Figure 4E*). In older age groups (70–100 years), saliency for P-wave duration and QRS duration remained high, while T-wave amplitude continued to show negative correlation (*r* = −0.17 to −0.24). Interestingly, HRV parameters showed a positive correlation with saliency in younger age groups (20–40 years), peaking at *r* = 0.11–0.13, but declining thereafter. Analysis of individual datasets (see Supplementary material online, *Figure S9*) confirmed the pooled findings.

### External subgroup analysis within the LEAD dataset

In the LEAD cohort (*Figure 5B*), median Δage (years, interquartile range) was significantly higher in smokers (5.19, −0.45 to 10.94), hypertension (3.86, −1.95 to 9.15), diabetes (0.48, −5.53 to 6.15), chronic obstructive pulmonary disease (COPD) (2.87, −2.05 to 8.10), and hypolipoproteinaemia (6.41, 0.56 to 12.15). Conversely, it was significantly lower in those with carotid calcification (−1.33, −6.72 to 3.56), myocardial infarction (MI) (−0.93, −5.09 to 4.03, *P* = 0.015), and stroke (−1.97, −6.50 to 3.14) vs. age-matched healthy controls (all *P* < 0.05).

**Figure 5:**
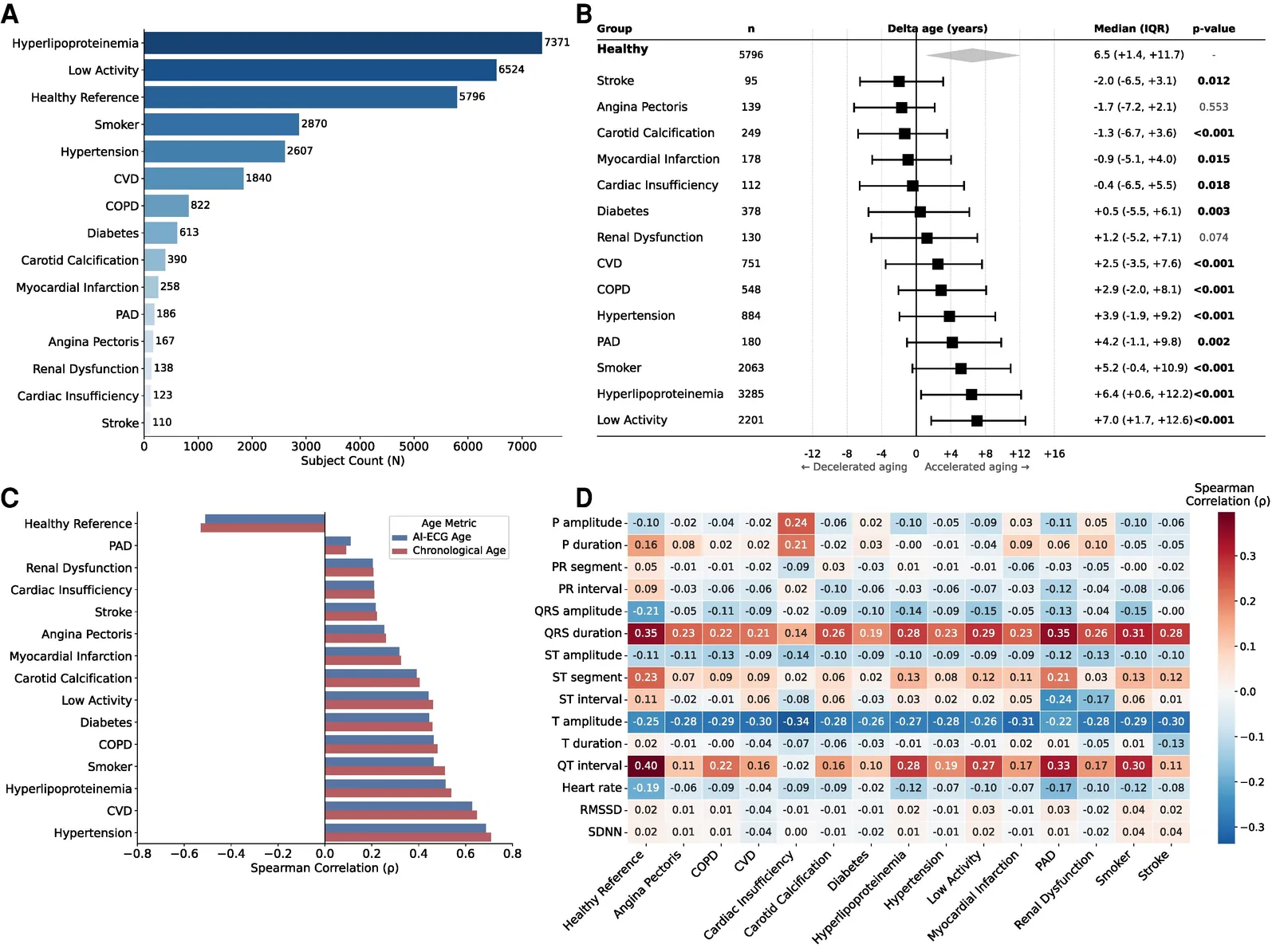
Impact of clinical comorbidities and lifestyle factors on artificial intelligence–electrocardiogram age predictions and model feature importance. (*A*) Horizontal bar chart summarizing the patient distribution of lifestyle factors and clinical comorbidities within the study cohort. (*B*) Forest plot of age- and sex-matched analyses depicting the shift in corrected Δage across clinical subgroups. The markers represent median values with interquartile ranges, indicating accelerated (positive) or decelerated (negative) predicted ageing relative to a baseline healthy reference group. (*C*) Grouped bar chart displaying Spearman rank correlations between the presence of specific comorbidities or lifestyle factors and continuous age metrics (artificial intelligence–electrocardiogramage and chronological age). (*D*) Heatmap of Spearman correlations revealing the relationship between physiological electrocardiogram features and model saliency across different disease subgroups, demonstrating how specific underlying pathologies modulate the model’s region-specific focus compared to healthy individuals.

Positive correlations were observed between the presence of comorbidities and AI–ECG age (*Figure 5C*), with correlation coefficients ranging from *r* = 0.21 to *r* = 0.70. Notably, correlations with chronological age were similarly strong (0.21–0.71), indicating that predictions track closely with actual age in most subgroups. Correlations of AI-ECG age and comorbidities of the public datasets aligned with these findings (see Supplementary material online, *Figure S10*).

Correlation analyses of ECG features with subgroups (*Figure 5D*) showed the most salient features were QRS duration and T-wave amplitude. QRS duration showed positive correlations with most disease states (0.14-0.35), with the strongest associations in COPD (0.22, *P* < 0.001), cardiovascular disease (CVD) (0.21, *P* < 0.001), and hypolipoproteinaemia (0.28, *P* < 0.001). T-wave amplitude exhibited consistently negative correlations (ranging from −0.22 to −0.34), most notably in stroke (−0.30, *P* < 0.001), cardiac insufficiency (−0.34, *P* < 0.001), and hypertension (−0.28, *P* < 0.001). ST segment and QT interval duration also showed positive correlations with disease presence, particularly in hypolipoproteinaemia and CVD. Heart rate and HRV parameters showed weak or non-significant correlations with most subgroups.

## Discussion

Our findings suggest that a transformer trained on globally diverse, heterogeneous ECG cohorts shows promise in comparison to state-of-the-art CNN models in predicting AI–ECG age across multiple public datasets (*Table 2*). Crucially, the transformer trained on global data achieved the highest correlation (*r* = 0.90, *P* < 0.001) with chronological age in the population-based LEAD cohort, surpassing even the LEAD-specific model (*r* = 0.88, *P* < 0.001). This suggests that a transformer model trained on global, heterogeneous data may generalize better than models trained on homogeneous cohorts.

In contrast, the LEAD-trained Transformer exhibited reduced performance and greater variance across datasets, reflecting a genuine reduction in discriminative capacity rather than statistical variation alone. Lacking exposure to pathological morphologies during training, it overfits to the ‘normal’ range of the LEAD cohort and fails to adapt to broader ECG phenotypes. This supports the idea that subclinical pathologies are not noise, but they contain physiological information enabling models to learn robust, age-sensitive features, even when not attributable to overt disease.

When disentangling heterogeneity from training size, it showed that shrinking the global model to the LEAD size raised pooled MAE on the public test sets from 8.6 to 11.5 years, indicating superior generalization stems partly from scale (see Supplementary material online, *Table S9*). Even at matched size, the heterogeneous model outperformed the LEAD model, indicating diverse phenotypes aid generalization regardless of volume. On the LEAD cohort, the Transformer (LEAD) performed best (MAE = 7.5 years), the size-matched model worse (13.2 years), and only the Transformer (Global) approached it (7.9 years), meaning that heterogeneity improves generalization across populations, but the global model needs full volume to match a cohort-specific model on that cohort’s own distribution. Hence, training on a single homogeneous cohort achieves high within-cohort accuracy but sacrifices transferability to differently distributed populations.

Notably, all models systematically underestimated AI-ECG age in older individuals (*Table 2*: mean Δage: −0.9 to −5.4 years) while overestimating it in healthy younger subjects (*Figure 5B*). This matches findings by Kim *et al*.,^28^ who reported overestimations of 8.3 and 6.2 years at ages 10–19 and 20–29, hypothesized by the authors due to heightened myocardial electrical activity and autonomic influences. Similarly, Evans *et al*.^29^ observed a more pronounced divergence with overestimations in hospitalized cardiovascular patients aged 20–29 by 14.4 years and underestimated in individuals aged 80–89 by 10.6 years, possibly reflecting survival bias and selection of less frail patients. Within our study, these biases stem primarily from training-data composition (see Supplementary material online, *Figures S12*, *S13*), which is skewed towards the fifth to seventh decade, with sparse representation under age 30. These systematic biases highlight the need for methodological refinements, such as age-stratified training, synthetic data augmentation,^30^ or adaptive thresholding.

Li *et al*.^31^ benchmarked CNNs against transformers across pretrained ECG models, finding CNNs more parameter-efficient with generally lower out-of-distribution loss, seemingly at odds with our finding that the global transformer out-generalized the CNN on the external cohort (*Table 2*). However, they evaluate diagnostic and demographic classification rather than age, pretrain on a single cohort (CODE), and assess transfer by linear probing on frozen features, whereas we address age regression, train end-to-end on multi-continental data, and evaluate on an unseen cohort. Since architecture rankings depend on task, scale, and data composition,^32^ our results extend their comparison to an unexamined regime rather than contradicting it, which aligns with their own conclusion that architecture–paradigm alignment matters more than scale.

The survival analysis (*Figure 3*) further underscores the clinical relevance of AI-ECG age, which other studies also found.^30,31^ In both, CODE-15 and SaMiTrop, individuals with a Δage ≥+8 years showed significantly increased mortality risk (HRs = 1.79–2.38), aligning with and often exceeding prior findings: Lima *et al*.^3^ and Chang *et al*.^33^ reported similar trends in the same cohorts, and a meta-analysis by Mossavarali *et al*.^7^ a pooled HR = 1.62 [1.49–1.77 95% CI] across multiple cohorts, suggesting that our observed effect may reflect cohort-specific or age-related amplification of risk. The age-residualized Cox analysis (*Table 5*) confirms Δage remains a strong, independent predictor of mortality after removing chronological age, showing robustness to calibration drift. Despite superior age estimation, the transformer did not outperform the CNN in prognosis: its discrimination was marginally lower (paired ΔC-index = −0.004), a clinically negligible gap. Thus, both architectures are prognostically equivalent and Δage–mortality reflects the ECG-age concept, not architecture-specific properties. Progressive adjustment for a normal-ECG flag and explicit ECG diagnoses attenuated the CODE-15 association only modestly (per-SD HR from 1.30 to 1.25; *P* < 0.001), indicating that Δage carries information beyond overt ECG disease, although residual confounding cannot be excluded and covariate availability differed between cohorts. The SaMiTrop estimates warrant caution since, as a small disease-specific cohort, its HRs are imprecise. This limited power also explains the apparent decelerated-ageing discrepancy, which was significantly protective in CODE-15 (HR = 0.85, *P* < 0.001) but non-significant and opposite in SaMiTrop (HR = 1.25, *P* = 0.377), most likely sampling variability rather than a contradictory biological effect.

The saliency analysis provided insights into which ECG regions most influenced the model’s age predictions. We have observed that the model assigns the highest attributions to the P-wave and PQ segment, particularly in lead V1 (*Figure 4A–C*), regions known to be sensitive to atrial activation and interatrial conduction abnormalities.^34,35^ Consistent with previous reports that age-estimation models concentrate attribution around the P-wave and atrial conduction,^36,37^ our results confirm and extend this pattern in a globally diverse cohort. This aligns with established literature linking P-wave morphology (e.g. P-wave duration, terminal force) to age-related atrial remodelling,^38^ atrial fibrillation risk,^39–41^ and left atrial enlargement.^36,42,43^ The model’s focus on early atrial events rather than late repolarization (T wave) or ventricular conduction (QRS) may indicate that the model relies on features associated with structural and electrophysiological remodelling associated with ageing, rather than late-stage ischaemic or hypertrophic changes. Age-stratified correlation analyses further refined this interpretation: saliency for P-wave and QRS duration peaks in midlife (30–50 years), coinciding with a period of active and clinically significant cardiac ageing and remodelling.^44–46^ In contrast, T-wave amplitude, which is often associated with repolarization abnormalities, shows a strong negative correlation with age, particularly in older cohorts (70–100 years), suggesting repolarization changes may become more prominent in advanced age.^44,47^ These age-dependent patterns are consistent with the hypothesis that the model relies on morphological and conduction features that co-vary with age, rather than an arbitrary age label. This hypothesis is consistent with the known pathophysiology of age-related cardiac remodelling. However, these are hypotheses requiring further investigation, and the retrospective nature of saliency analysis must be acknowledged.

Subgroup analyses revealed that AI–ECG age systematically reflects underlying cardiovascular and metabolic burdens (*Figure 5*). Conditions such as smoking, hypertension, and hypolipoproteinaemia, known accelerators of ageing, were associated with significant positive Δage, suggesting the model is sensitive to systemic stressors that manifest as electrophysiological remodelling. This aligns with prior studies linking increased Δage to lifestyle factors such as smoking or low physical activity,^48^ and to higher risk of developing comorbidities,^12^ reinforcing the model’s sensitivity to cumulative physiological strain. Counterintuitively, Δage was significantly lower in stroke, MI, and carotid calcification, conditions correlating with age, and their cohorts were older (see Supplementary material online, *Figure S3*). The reduction persisted against age- and sex-matched controls, ruling out age bias and indicating a disease-specific effect. It may reflect focal ECG changes from structural disease that do not align with the continuous ageing signatures the network captures, and normalization of age-related traits by secondary-prevention medication. Δage may therefore underestimate ageing in established structural disease; disease-aware calibration and medication-adjusted validation are needed.

Several limitations remain. First, all analyses are retrospective, based on linked ECG and mortality data. The model’s ability to predict future ageing trajectories or mortality risk remains untested. Prospective validation in longitudinal cohorts is essential to assess its predictive power for incident cardiovascular events or mortality. Moreover, longitudinal mortality data existed only for CODE-15 and SaMiTrop, which both contributed to model development. Although all survival-analysis Δage values were out-of-fold predictions, the Δage–mortality association was not tested in an independent cohort. Prognostic findings should therefore be read as supporting the plausibility of Δage as a biological-ageing marker within these cohorts, not as established clinical utility, which requires dedicated external validation. Second, saliency maps indicate regions of high attribution but do not establish causal or mechanistic relationships: a recent study found they do not identify which signal property drives a prediction, are prone to confirmation bias, can retain attributions even when model weights are randomized, and have limited clinical utility.^49^ In particular, the gradient method used here provides a first-order, single-method attribution estimate that is susceptible to gradient saturation and input noise. The attribution pattern was nonetheless reproducible across all eleven contributing datasets and across age strata (*Figure 4E*; Supplementary material online, *Figure S9*), arguing against a cohort-specific or purely noise-driven artefact. Therefore, these attributions should not be interpreted as evidence that the model ‘understands’ atrial remodelling but rather serve as exploratory hypotheses for biological validation. To further validate biological plausibility beyond attribution heatmaps, future work should incorporate ablation studies, counterfactual analysis, or feature-level causal inference, and convergent, multi-method attribution (e.g. Integrated Gradients, SmoothGrad, or occlusion testing) to establish robustness to methodological choices and noise.

Third, our Cox model adjusted for sex and age, but not for other clinically relevant confounders since such data were not publicly available for these datasets. These variables may independently influence both AI-ECG age and mortality risk, potentially confounding the observed association between Δage and hazard. Future analyses should incorporate these covariates to better isolate the effect of age acceleration.

Finally, AI-ECG age is a model-derived prediction, not a biological age measure (e.g. epigenetic clocks or telomere length). While it correlates with chronological age, it remains unclear whether it reflects biological ageing or merely chronological proxies.

As a future route, although not examined in this study and currently only receiving limited direct empirical support,^50,51^ it remains possible that integrating AI-ECG–derived age with biomarkers of cardiac cellular senescence could contribute to earlier identification of cardiovascular ageing since senescence-related changes across cardiac and vascular cell types may influence electrophysiology and could precede clinically apparent cardiac ageing, potentially giving rise to subtle ECG alterations.^52,53^ Although this idea has been previously postulated,^54^ further investigation is required to evaluate whether combining digital and biological markers of senescence has utility for risk stratification or mechanistic insight in age-related CVD.

Additionally, while traditional machine learning methods that have been used in the last decade^2^ offer comparable performance, and such as the models developed by Lindow *et al*.,^11,55^ deep learning models provide greater flexibility and predictive power for ECG-based age estimation. For example, the model developed by Lindow *et al*.^11,55^ requires sinus rhythm due to its reliance on P-wave duration, excluding patients with arrhythmias, whereas deep learning can generalize across diverse rhythms. It also depends on predefined features and loses high-fidelity information from longer recordings, while deep learning extracts subtle, latent patterns directly from raw ECG signals.

Recently, self-supervised ECG foundation models (FMs)^56^ have matched a strong supervised baseline with nine times fewer labelled examples via a reusable encoder, its lowest age-estimation error coming from a transformer.^57^ Yet only three of eight FMs beat a well-tuned supervised baseline on age regression, the largest performed worst, and rankings were evaluation-dependent.^57^ Supervised models thus remain competitive, and our MAEs (*Table 2*) fall within the range that benchmark reported for both architectures,^57^ albeit on a different cohort. Self-supervised pretraining is nonetheless promising where annotated data are scarce.

This directly relates to future potential: to fully realize the promise of AI-ECG age, research should shift towards real-world deployment, e.g. on wearable devices, to enable scalable, proactive monitoring of cardiovascular ageing. This aligns with emerging efforts using photoplethysmography data from smartwatches^58^ or from the UK Biobank and MIMIC-III datasets^59^ as well as single lead ECGs from patches,^60^ suggesting a future where digital phenotyping complements traditional diagnostics to move from reactive to preventive cardiovascular medicine.

To summarize, this study advances the field by demonstrating that transformer models trained on globally diverse, heterogeneous ECGs are more generalizable than models trained on homogenous cohorts, if trained on sufficient data. The model’s focus on P-wave morphology and its correlation with known ageing and disease mechanisms provides insights into its predictions. We believe that, when combined with broader digital phenotyping and deployed in real-world settings, AI–ECG age may offer a novel way to identify individuals with accelerated cardiovascular ageing and associated risk.

## Lead author biography


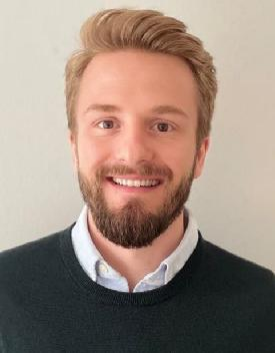


Laurenz Berger completed his master’s degree in biomedical engineering at the Technical University of Vienna, Austria and is currently pursuing his PhD in biomedical engineering at the Center for Medical Physics and Biomedical Engineering at the Medical University of Vienna, Austria. His research focuses on advancing data analysis techniques for wearable devices, with applications in cardiovascular engineering and surgical innovation. A key aspect of his work involves leveraging deep learning, particularly generative artificial intelligence, to drive innovation in these fields.

## Supplementary Material

ztag149_Supplementary_Data

## Data Availability

The public datasets used in this study are publicly available to everyone in the following repositories https://zenodo.org/records/4916206, https://zenodo.org/records/4905618, https://physionet.org/content/challenge-2021/1.0.3/, https://physionet.org/content/ludb/1.0.1/. The Lung, hEart, sociAl, boDy (LEAD) dataset is restricted due to ethical limitations.

## Code availability

The code for the pretrained CNN for the model training and evaluation is available at the github repository https://github.com/antonior92/ecg-age-prediction. The code for the original architecture of the multivariate time series transformer is available at the github repository https://github.com/gzerveas/mvts_transformer. The code of this paper is available at the github repository https://github.com/lbp92/global-ecg-age-transformer.
