# Supplementary material for: Explainable age prediction using a transformer trained on diverse global electrocardiograms
Source: Eur Heart J Digit Health. 2026 Sep 23;7(8):ztag149. doi: 10.1093/ehjdh/ztag149 (PMC13652029; doi:10.1093/ehjdh/ztag149)
Supplement: Supplementary file 1 [file ztag149_supplementary_data.docx]

Explainable Age Prediction Using a Transformer Trained on Diverse Global Electrocardiograms

Supplement

Contents

[**Databases Information**	3](#_Toc234827979)

[**Modelling Parameters and Hyperparameter Search**	3](#_Toc234827980)

[**ECG Delineation**	4](#_Toc234827981)

[**Analysis of ECGs excluded from the explainability analysis**	4](#_Toc234827982)

[**ECG Parameter Calculation Validation**	5](#_Toc234827983)

[**Table S1**	5](#_Toc234827984)

[**Table S2**	7](#_Toc234827985)

[**Table S3**	9](#_Toc234827986)

[**Table S4**	11](#_Toc234827987)

[**Table S5**	11](#_Toc234827988)

[**Table S6**	12](#_Toc234827989)

[**Table S7**	12](#_Toc234827990)

[**Table S8**	12](#_Toc234827991)

[**Figure S1**	15](#_Toc234827992)

[**Figure S2**	16](#_Toc234827993)

[**Figure S3**	17](#_Toc234827994)

[**Figure S4**	17](#_Toc234827995)

[**Figure S5**	18](#_Toc234827996)

[**Figure S6**	19](#_Toc234827997)

[**Figure S7**	19](#_Toc234827998)

[**Figure S8**	20](#_Toc234827999)

[**Figure S9**	21](#_Toc234828000)

[**Figure S10**	21](#_Toc234828001)

[**Figure S11**	22](#_Toc234828002)

[**Figure S12**	23](#_Toc234828003)

[**Figure S13**	23](#_Toc234828004)

# **Databases Information**

The Code-15 and the SaMiTrop datasets were downloaded from [https://zenodo. org/records/4916206](https://zenodo.org/records/4916206) and [https://zenodo.org/records/490561](https://zenodo.org/records/4905618)8, respectively. These two datasets were already preprocessed by the authors. The CPSC, CPSC extra, St. Petersburg INCART, PTB-XL, PTB, Georgia, Ningbo, Shaoxing datasets are part of the Physionet Challenge 2021 and were downloaded from [https://physionet.org/content/ challenge-2021/1.0.3/](https://physionet.org/content/challenge-2021/1.0.3/). The LUDB dataset was and downloaded from <https://physionet.org/content/ludb/1.0.1/>. For each individual in the Code-15 dataset, additional data was available indicating whether they had first-degree atrioventricular block, right bundle branch block, left bundle branch block, sinus bradycardia, sinus tachycardia or atrial fibrillation. For the individuals in the SaMiTrop dataset no information about additional conditions present was supplied. Nevertheless, a flag was included indicating if the individual had a "normal ECG". 18% of the individuals had a normal ECG. Additionally, information was supplied whether the individual died within the follow-up period. If the individual died the information within period was available as well. For the datasets being part of the PhysioNet Challenge (Shaoxing, CPSC, CPSC extra, Georgia, Ningbo, PTB, PTB-XL, St. Petersburg INCART) besides age and sex additional information on certain conditions were present. More specifically for 133 conditions with the corresponding Systematized Nomenclature of Medicine Clinical Terms (SNOMED CT) Codes are provided. For 22 individuals the sex was unknown. Many conditions only appear in certain datasets. Some conditions appear in all the datasets only once. Sinus rhythm, sinus bradycardia, T wave abnormal, sinus tachycardia and atrial flutter are the most common conditions observed in these datasets. For the LUDB besides sex and age additional information was provided within several categories. The categories include rhythm, electric axis of the heart, conduction abnormalities, extrasystoles, hypertrophies, cardiac pacing, ischemia, non-specific repolarization abnormalities and other states. In more than 50 % of the individuals had left ventricular hypertrophy or/and left atrial hypertrophy.

# **Modelling Parameters and Hyperparameter Search**

As our main model a multivariate time series transformer was implemented as proposed in Zerveas et al. and optimized using five-fold cross-validation, for which all public datasets were divided into five equal-sized sets. In each iteration, three sets were used for training, one for validation, and one was reserved for testing. To ensure that testing primarily assessed generalization on external datasets, two considerations were made during dataset partitioning: First, individual datasets were not split across sets, except the CODE-15 dataset necessitating division for balanced sampling due to its size and second, datasets with similar origin, such as the PTB and PTB-XL datasets, were assigned to the same set. A random search hyperparameter optimization was conducted to explore the search space followed by 20 additional iterations using a tree-structured Parzen estimator algorithm, which efficiently searches across continuous, discrete, and conditional parameters by modeling promising regions of the space. Activation functions included Rectified Linear Units (ReLU) and Gaussian Error Linear Units (GELU). The batch size was set to 64, and the RAdam optimizer was used. The first transformer layer was a 1D convolutional neural network (CNN) layer, with kernel size, padding, and stride optimized during the search. Additionally, the initial learning rate, positional encoding, and dropout rate were optimized. Training was performed for a maximum of 128 epochs, with early stopping applied if no improvement was observed on the validation set for 10 consecutive epochs. Random seed=1337 was used for dataset splitting and random seed=42 for training. For all models the root mean squared error between AI-ECG age and chronological age was used as training loss metric. The best model was determined based on the mean absolute error between AI-ECG age and chronological age. Analyses were performed on a Nvidia H100 GPU (80GB VRAM), Intel Xeon Platinum 8480C (16 cores, 128GB RAM), using Python 3.10.

# **ECG Delineation**

To ensure the robustness of the saliency analysis, we decoupled the segment extraction logic from the direct, often unstable outputs of the automated deep learning delineator. Instead of relying on symmetrically predicted waveform onsets and offsets, we developed a beat-centric, R-peak anchored extraction algorithm.

First, structural delineations (P-waves, QRS-complexes, and T-waves) were extracted globally and sanitized to remove anomalous predictions (e.g., duplicate morphological features within $\leq50$ ms) using neurokit2 Python package. Next, individual cardiac cycles were constructed chronologically around each delineated R-peak. The R-R interval ($RR_{n}$) was utilized as a local benchmark to define physiologically plausible search limits for associated features. Specifically, for an R-peak $R_{i}$:

1. A true QRS-complex was established by locating the nearest QRS onset $\leq120$ ms prior to $R_{i}$, and an offset $\leq200$ ms post $R_{i}$.
2. The corresponding P-wave was defined dynamically by identifying the latest P-onset and P-offset occurring before the localized QRS onset, bounded entirely within $0.5\times RR_{n}$ to prohibit erroneous pairing with the prior cardiac cycle.
3. The T-wave was analogously defined by searching for onsets and offsets bounding the terminal tail of the localized QRS complex, constrained within $0.7\times RR_{n}$.

Once a validated cardiac cycle array—complete with onset and offset indices—was resolved for a given beat, morphological and attribution metrics were extracted. To mitigate model hallucination artifacts, sub-segments demonstrating negligible maximum normalized absolute amplitudes ($\left< 0.02 \right.$) across the specified integration window were immediately discarded. Finally, temporal durations, normalized signal extrema, peak-to-peak amplitudes, mathematical integrals (AUC), and mean gradient slopes were calculated over each rigorously validated segment, alongside corresponding localized saliency magnitudes.

# **Analysis of ECGs excluded from the explainability analysis**

Analysis of ECGs excluded from the explainability analysis ECGs were excluded from the saliency-based explainability analysis for one of two reasons: (1) delineation failure, in which case the automated delineation algorithm did not produce a result for the recording; or (2) out-of-range parameters, in which case the delineation succeeded but one or more derived physiological parameters (e.g. interval durations, amplitudes) fell outside pre-specified physiologically plausible bounds. ECGs passing both criteria constitute the included population. To assess whether exclusion introduced systematic bias, included and excluded recordings were compared across datasets on the following characteristics: age (years), sex and, where annotation metadata were available, ECG rhythm labels. Continuous variables (age) are presented as mean ± standard deviation and compared between included and excluded recordings using Welch’s two-sample t-test; the standardized mean difference (Cohen’s d) is additionally reported for the pooled comparison. Binary variables (sex, ECG findings, comorbidities) are presented as counts with percentages of the respective group and compared using the χ² test. Excluded ECGs were analyzed in aggregate (all excluded vs. included) as the primary comparison and additionally broken down by exclusion reason (delineation failure vs. out-of-range parameters) for descriptive purposes. A pooled comparison aggregating all twelve datasets was performed alongside per-dataset analyses. Statistical significance was defined as p < 0.05; given the descriptive nature of this analysis and the very large sample size, no correction for multiple comparisons was applied and emphasis is placed on effect sizes rather than p-values alone.

Of 449,631 ECGs across twelve datasets, 370,479 (82.4%) passed both quality-control criteria and were included in the explainability analysis, while 79,152 (17.6%) were excluded. Exclusions were almost entirely attributable to out-of-range derived parameters (77,189; 97.5% of exclusions; 17.2% of all ECGs); outright delineation failure was rare (1,963; 2.5% of exclusions; 0.4% of all ECGs). The exclusion rate varied across datasets, from 14.0% (LUDB) to 39.2% (INCART, n = 74), and was 15.8% in CODE-15, which contributed 76.9% of all recordings and therefore dominates the pooled estimates (Table S8).

Pooled across datasets, excluded recordings were modestly older than included recordings (56.8 ± 19.6 vs. 53.4 ± 19.8 years; Welch’s t-test p < 0.001; Cohen’s d = 0.17) and more frequently male (51.4% vs. 39.7%; χ² p < 0.001). The male over-representation among excluded ECGs was consistent, observed in 10 of 11 datasets with sex annotation, whereas the age difference, although significant in the pool, was small and inconsistent in direction at the dataset level (excluded older in 8 of 12 datasets; Supplementary Table S8). Excluded ECGs were systematically enriched for rhythm and conduction abnormalities and depleted of normal sinus rhythm. Normal sinus rhythm was less prevalent among excluded recordings in 9 of 10 datasets with rhythm labels (e.g. PTB-XL, 86.8% vs. 68.4%; Ningbo, 22.6% vs. 4.1%; Shaoxing, 24.0% vs. 3.0%). Conversely, sinus bradycardia (more frequent in excluded recordings in 7 of 7 datasets where present; mean difference +8.7 percentage points), left ventricular hypertrophy (7 of 7), atrial fibrillation (7 of 9), atrial flutter (6 of 7) and left bundle-branch block (6 of 7) were over-represented among excluded recordings. Across all dataset-level findings present at ≥ 1% prevalence, 93 of 139 (67%) were more prevalent among excluded than among included recordings. The small subgroup excluded for delineation failure showed the same pattern more sharply, being strongly enriched for wide-complex and conduction abnormalities (e.g. first-degree atrioventricular block, left bundle-branch block, left-axis deviation, atrial flutter; Table S9 and Figure S11). These patterns are mechanistically expected: ECGs with extreme heart rates or abnormal, wide or low amplitude complexes are both harder to delineate and more likely to yield interval or amplitude measurements outside physiologically plausible bounds. Exclusion therefore acted as a signal-quality and plausibility filter that correlated with — but was not defined by — the presence of arrhythmia and conduction disease. Supplementary Discussion — implications for the explainability analysis Exclusion was non-random: the retained population was modestly younger and more often female, and was enriched for normal sinus rhythm relative to excluded recordings. Three considerations, however, bound the likely impact on the saliency interpretation. First, the demographic differences were small in magnitude (age Cohen’s d = 0.17; sex difference ≈ 12 percentage points) despite reaching significance at this sample size. Second, exclusion was driven by the signal quality and physiological plausibility of the measured parameters rather than by the diagnostic labels that the saliency analysis targets; the included set therefore comprises higher-quality signals in which delineation-based attributions are more reliable. Third, every rhythm and morphology category remained represented in the included population in absolute numbers sufficient for class-level saliency estimation (several thousand atrial fibrillation, myocardial infarction and bundle-branch-block recordings were retained). We nonetheless acknowledge as a limitation that the most extreme or low-quality morphologies are under-represented among included recordings, so the saliency maps may modestly under-weight features specific to those presentations; this should be considered when generalizing the explainability findings to the most abnormal ECGs.

# **ECG Parameter Calculation Validation**

ECG-derived physiological parameters were filtered using predefined plausibility bounds informed by standard clinical ranges. Briefly, each dataset file containing segment-level features was loaded, and rows were retained only if all available parameters lay within expected limits. Thresholds were defined per feature and, where appropriate, per ECG segment (e.g., P wave, QRS complex, ST segment, T wave). For segment-specific checks, a row was excluded only when the row’s segment matched the target segment and the feature value was outside the allowed interval; for global features, out-of-range values triggered exclusion regardless of segment. Applied bounds included durations (e.g., P-wave, QRS, ST, T-wave), heart rate, peak-to-peak amplitudes, extrema, RR-interval variability metrics (SDNN, RMSSD), and slope-based descriptors.

# **Table S1**

**The following table shows the SNOMED CT Codes for the datasets which are included in the PhysioNet Challenge.** The numbers indicate how many individuals had the given condition in the respective dataset.

| **SNOMED CT** | **Abbreviation** | **CPSC** | **CPSC extra** | **St. Petersburg INCART** | **PTB** | **PTB-XL** | **Georgia** | **Shaoxing** | **Ningbo** | **Total** |
| --- | --- | --- | --- | --- | --- | --- | --- | --- | --- | --- |
| 233892002 | AAR | 0 | 0 | 0 | 0 | 0 | 0 | 0 | 16 | 16 |
| 251173003 | AB | 0 | 0 | 3 | 0 | 0 | 0 | 3 | 0 | 6 |
| 251187003 | AED | 0 | 0 | 0 | 0 | 0 | 0 | 0 | 17 | 17 |
| 164889003 | AF | 1221 | 153 | 2 | 15 | 1514 | 570 | 1780 | 0 | 5255 |
| 195080001 | AFAFL | 0 | 39 | 0 | 0 | 0 | 2 | 0 | 0 | 41 |
| 164890007 | AFL | 0 | 54 | 0 | 1 | 73 | 186 | 445 | 7615 | 8374 |
| 195126007 | AH | 0 | 2 | 0 | 0 | 0 | 60 | 0 | 0 | 62 |
| 61277005 | AIVR | 0 | 0 | 0 | 0 | 0 | 0 | 0 | 14 | 14 |
| 426664006 | AJR | 0 | 0 | 0 | 0 | 0 | 19 | 0 | 12 | 31 |
| 251139008 | ALR | 0 | 0 | 0 | 0 | 0 | 12 | 0 | 0 | 12 |
| 57054005 | AMI | 0 | 0 | 6 | 0 | 0 | 0 | 0 | 49 | 55 |
| 413444003 | AMIs | 0 | 1 | 0 | 0 | 0 | 1 | 0 | 0 | 2 |
| 251268003 | AP | 0 | 0 | 0 | 0 | 0 | 52 | 0 | 0 | 52 |
| 106068003 | ARH | 0 | 0 | 0 | 0 | 0 | 0 | 0 | 215 | 215 |
| 713422000 | ATach | 0 | 15 | 0 | 0 | 0 | 28 | 121 | 176 | 340 |
| 233917008 | AVB | 0 | 5 | 0 | 0 | 0 | 74 | 166 | 78 | 323 |
| 50799005 | AVD | 0 | 0 | 0 | 0 | 0 | 0 | 0 | 59 | 59 |
| 29320008 | AVJR | 0 | 6 | 0 | 0 | 0 | 0 | 0 | 139 | 145 |
| 251166008 | AVNRT | 0 | 0 | 0 | 0 | 0 | 0 | 16 | 0 | 16 |
| 233897008 | AVRT | 0 | 0 | 0 | 0 | 0 | 0 | 8 | 18 | 26 |
| 54329005 | AnMI | 0 | 62 | 0 | 0 | 354 | 0 | 0 | 57 | 473 |
| 426434006 | AnMIs | 0 | 0 | 0 | 0 | 44 | 281 | 0 | 0 | 325 |
| 6374002 | BBB | 0 | 0 | 1 | 20 | 0 | 116 | 0 | 385 | 522 |
| 251170000 | BPAC | 0 | 2 | 3 | 0 | 0 | 0 | 0 | 62 | 67 |
| 418818005 | BRU | 0 | 0 | 0 | 0 | 0 | 0 | 0 | 5 | 5 |
| 74615001 | BTS | 0 | 1 | 1 | 0 | 0 | 0 | 0 | 0 | 2 |
| 426627000 | Brady | 0 | 271 | 11 | 0 | 0 | 6 | 0 | 7 | 295 |
| 426749004 | CAF | 0 | 1 | 0 | 0 | 0 | 0 | 0 | 0 | 1 |
| 251199005 | CCR | 0 | 0 | 0 | 0 | 0 | 0 | 162 | 0 | 162 |
| 698247007 | CD | 0 | 0 | 0 | 16 | 0 | 0 | 0 | 0 | 16 |
| 27885002 | CHB | 0 | 27 | 0 | 0 | 16 | 8 | 1 | 75 | 127 |
| 53741008 | CHD | 0 | 0 | 16 | 21 | 0 | 0 | 0 | 0 | 37 |
| 204384007 | CIAHB | 0 | 0 | 0 | 2 | 0 | 0 | 0 | 0 | 2 |
| 733534002 | CLBBB | 0 | 0 | 0 | 0 | 0 | 0 | 0 | 213 | 213 |
| 413844008 | CMI | 0 | 161 | 0 | 0 | 0 | 0 | 0 | 0 | 161 |
| 251198002 | CR | 0 | 0 | 0 | 0 | 0 | 0 | 76 | 0 | 76 |
| 713427006 | CRBBB | 0 | 113 | 0 | 0 | 542 | 28 | 0 | 1096 | 1779 |
| 61721007 | CVCL/CCVCL | 0 | 0 | 0 | 0 | 0 | 0 | 0 | 653 | 653 |
| 82226007 | DIB | 0 | 1 | 0 | 0 | 0 | 0 | 0 | 0 | 1 |
| 428417006 | ERe | 0 | 0 | 0 | 0 | 0 | 140 | 22 | 344 | 506 |
| 13640000 | FB | 0 | 0 | 7 | 0 | 0 | 0 | 2 | 114 | 123 |
| 164942001 | FQRS | 0 | 0 | 0 | 0 | 0 | 0 | 3 | 0 | 3 |
| 84114007 | HF | 0 | 0 | 0 | 7 | 0 | 0 | 0 | 0 | 7 |
| 251259000 | HTV | 0 | 1 | 0 | 0 | 0 | 0 | 0 | 0 | 1 |
| 368009 | HVD | 0 | 0 | 0 | 6 | 0 | 0 | 0 | 0 | 6 |
| 270492004 | IAVB | 722 | 106 | 0 | 0 | 797 | 769 | 247 | 893 | 3534 |
| 251200008 | ICA | 0 | 0 | 0 | 0 | 156 | 0 | 0 | 0 | 156 |
| 195042002 | IIAVB | 0 | 21 | 0 | 0 | 14 | 23 | 8 | 58 | 124 |
| 426183003 | IIAVBII | 0 | 0 | 0 | 0 | 0 | 0 | 0 | 7 | 7 |
| 425419005 | IIs | 0 | 0 | 0 | 0 | 219 | 451 | 0 | 0 | 670 |
| 251120003 | ILBBB | 0 | 42 | 0 | 0 | 77 | 86 | 0 | 6 | 211 |
| 49260003 | IR | 0 | 0 | 2 | 0 | 0 | 0 | 0 | 0 | 2 |
| 713426002 | IRBBB | 0 | 86 | 0 | 0 | 1118 | 407 | 0 | 246 | 1857 |
| 704997005 | ISTD | 0 | 1 | 0 | 0 | 0 | 0 | 0 | 0 | 1 |
| 426995002 | JE | 0 | 4 | 0 | 0 | 0 | 5 | 15 | 60 | 84 |
| 251164006 | JPC | 0 | 2 | 0 | 0 | 0 | 0 | 1 | 10 | 13 |
| 426648003 | JTach | 0 | 2 | 0 | 0 | 0 | 4 | 0 | 24 | 30 |
| 253352002 | LAA | 0 | 0 | 0 | 0 | 0 | 72 | 0 | 0 | 72 |
| 39732003 | LAD | 0 | 0 | 0 | 0 | 5146 | 940 | 382 | 1163 | 7631 |
| 6.7741E+13 | LAE | 0 | 1 | 0 | 0 | 427 | 870 | 0 | 1 | 1299 |
| 446813000 | LAH | 0 | 40 | 0 | 0 | 0 | 0 | 0 | 8 | 48 |
| 445118002 | LAnFB | 0 | 0 | 0 | 0 | 1626 | 180 | 0 | 380 | 2186 |
| 164909002 | LBBB | 236 | 38 | 0 | 0 | 536 | 231 | 205 | 35 | 1281 |
| 425623009 | LIs | 0 | 0 | 0 | 0 | 142 | 903 | 0 | 0 | 1045 |
| 445211001 | LPFB | 0 | 0 | 0 | 0 | 177 | 25 | 0 | 5 | 207 |
| 164947007 | LPR | 0 | 0 | 0 | 0 | 340 | 0 | 12 | 40 | 392 |
| 251146004 | LQRSV | 0 | 0 | 0 | 0 | 182 | 374 | 249 | 794 | 1599 |
| 111975006 | LQT | 0 | 4 | 0 | 0 | 118 | 1391 | 57 | 337 | 1907 |
| 164873001 | LVH | 0 | 158 | 10 | 0 | 2359 | 1232 | 15 | 632 | 4406 |
| 55827005 | LVHV | 0 | 0 | 0 | 0 | 0 | 0 | 1295 | 4106 | 5401 |
| 370365005 | LVS | 0 | 1 | 0 | 0 | 0 | 0 | 0 | 0 | 1 |
| 164865005 | MI | 0 | 376 | 9 | 368 | 5261 | 7 | 40 | 83 | 6144 |
| 164861001 | MIs | 0 | 384 | 0 | 0 | 2175 | 0 | 0 | 0 | 2559 |
| 54016002 | MoI | 0 | 0 | 3 | 0 | 0 | 0 | 6 | 25 | 34 |
| 698252002 | NSIVCB | 0 | 4 | 1 | 0 | 789 | 203 | 235 | 536 | 1768 |
| 426783006 | NSR | 918 | 4 | 0 | 80 | 18092 | 1752 | 1826 | 6299 | 28971 |
| 428750005 | NSSTTA | 0 | 1290 | 0 | 0 | 381 | 1883 | 1158 | 0 | 4712 |
| 164867002 | OldMI | 0 | 1168 | 0 | 0 | 0 | 0 | 0 | 0 | 1168 |
| 284470004 | PAC | 616 | 73 | 3 | 0 | 398 | 639 | 258 | 1054 | 3041 |
| 282825002 | PAF | 0 | 0 | 1 | 1 | 0 | 0 | 0 | 0 | 2 |
| 251205003 | PPW | 0 | 0 | 0 | 0 | 0 | 0 | 0 | 106 | 106 |
| 10370003 | PR | 0 | 3 | 0 | 0 | 296 | 0 | 0 | 1182 | 1481 |
| 365413008 | PRWP | 0 | 0 | 0 | 0 | 0 | 0 | 0 | 638 | 638 |
| 67198005 | PSVT | 0 | 0 | 3 | 0 | 24 | 0 | 0 | 0 | 27 |
| 427172004 | PVC | 0 | 188 | 0 | 0 | 0 | 0 | 0 | 1091 | 1279 |
| 425856008 | PVT | 0 | 0 | 15 | 0 | 0 | 0 | 0 | 109 | 124 |
| 164912004 | PWC | 0 | 0 | 0 | 0 | 0 | 0 | 95 | 47 | 142 |
| 164917005 | QAb | 0 | 1 | 0 | 0 | 548 | 464 | 235 | 828 | 2076 |
| 253339007 | RAAb | 0 | 0 | 0 | 0 | 0 | 14 | 0 | 0 | 14 |
| 47665007 | RAD | 0 | 1 | 0 | 0 | 343 | 83 | 215 | 638 | 1280 |
| 314208002 | RAF | 0 | 0 | 0 | 2 | 0 | 0 | 0 | 0 | 2 |
| 446358003 | RAH | 0 | 18 | 0 | 0 | 99 | 0 | 3 | 33 | 153 |
| 6.7751E+13 | RAHV | 0 | 0 | 0 | 0 | 0 | 0 | 8 | 28 | 36 |
| 164921003 | RAb | 0 | 1 | 0 | 0 | 0 | 10 | 0 | 0 | 11 |
| 59118001 | RBBB | 1857 | 1 | 2 | 0 | 0 | 542 | 454 | 195 | 3051 |
| 89792004 | RVH | 0 | 20 | 0 | 0 | 126 | 86 | 4 | 106 | 342 |
| 427393009 | SA | 0 | 11 | 2 | 0 | 772 | 455 | 0 | 2550 | 3790 |
| 17366009 | SAAWR | 0 | 0 | 0 | 0 | 0 | 0 | 7 | 0 | 7 |
| 65778007 | SAB | 0 | 9 | 0 | 0 | 0 | 0 | 0 | 5 | 14 |
| 5609005 | SARR | 0 | 0 | 0 | 0 | 0 | 0 | 0 | 33 | 33 |
| 426177001 | SB | 0 | 45 | 0 | 0 | 637 | 1677 | 3889 | 12670 | 18918 |
| 60423000 | SND | 0 | 0 | 2 | 0 | 0 | 0 | 0 | 0 | 2 |
| 49578007 | SPRI | 0 | 3 | 0 | 0 | 0 | 2 | 0 | 23 | 28 |
| 77867006 | SQT | 0 | 1 | 0 | 0 | 0 | 0 | 0 | 2 | 3 |
| 55930002 | STC | 0 | 1 | 0 | 0 | 770 | 6 | 0 | 4232 | 5009 |
| 429622005 | STD | 869 | 57 | 4 | 0 | 1009 | 38 | 402 | 1266 | 3645 |
| 164931005 | STE | 220 | 66 | 4 | 0 | 28 | 134 | 176 | 0 | 628 |
| 164930006 | STIAb | 0 | 481 | 2 | 0 | 0 | 992 | 2 | 799 | 2276 |
| 427084000 | STach | 0 | 303 | 11 | 1 | 826 | 1261 | 1568 | 5687 | 9657 |
| 251168009 | SVB | 0 | 0 | 1 | 0 | 0 | 0 | 0 | 0 | 1 |
| 63593006 | SVPB | 0 | 53 | 4 | 0 | 157 | 1 | 0 | 9 | 224 |
| 426761007 | SVT | 0 | 3 | 1 | 0 | 27 | 32 | 587 | 137 | 787 |
| 164934002 | TAb | 0 | 22 | 0 | 0 | 2345 | 2306 | 1876 | 5167 | 11716 |
| 266257000 | TIA | 0 | 0 | 7 | 0 | 0 | 0 | 0 | 0 | 7 |
| 59931005 | TInv | 0 | 5 | 1 | 0 | 294 | 812 | 157 | 2720 | 3989 |
| 251223006 | TPW | 0 | 0 | 0 | 0 | 0 | 0 | 0 | 215 | 215 |
| 164937009 | UAb | 0 | 1 | 0 | 0 | 0 | 0 | 22 | 114 | 137 |
| 11157007 | VBig | 0 | 5 | 9 | 0 | 82 | 2 | 3 | 0 | 101 |
| 164884008 | VEB | 700 | 0 | 49 | 0 | 1154 | 41 | 0 | 0 | 1944 |
| 75532003 | VEsB | 0 | 3 | 1 | 0 | 0 | 0 | 7 | 49 | 60 |
| 81898007 | VEsR | 0 | 1 | 0 | 0 | 0 | 1 | 0 | 96 | 98 |
| 164896001 | VF | 0 | 10 | 0 | 25 | 0 | 3 | 0 | 59 | 97 |
| 111288001 | VFL | 0 | 1 | 0 | 0 | 0 | 0 | 0 | 7 | 8 |
| 266249003 | VH | 0 | 5 | 0 | 13 | 30 | 71 | 0 | 0 | 119 |
| 17338001 | VPB | 0 | 8 | 0 | 0 | 0 | 357 | 294 | 0 | 659 |
| 195060002 | VPEx | 0 | 6 | 0 | 0 | 0 | 2 | 12 | 0 | 20 |
| 251266004 | VPP | 0 | 0 | 0 | 0 | 0 | 46 | 0 | 0 | 46 |
| 251182009 | VPVC | 0 | 0 | 23 | 0 | 0 | 0 | 0 | 0 | 23 |
| 164895002 | VTach | 0 | 1 | 1 | 10 | 0 | 0 | 0 | 0 | 12 |
| 251180001 | VTrig | 0 | 4 | 4 | 0 | 20 | 1 | 8 | 0 | 37 |
| 195101003 | WAP | 0 | 0 | 0 | 0 | 0 | 7 | 2 | 0 | 9 |
| 74390002 | WPW | 0 | 0 | 4 | 2 | 80 | 2 | 4 | 68 | 160 |
| 164951009 | abQRS | 0 | 0 | 0 | 0 | 3389 | 0 | 0 | 0 | 3389 |

# **Table S2**

**The following table shows the categories and conditions that are included in the LUDB dataset.** The numbers indicate how many individuals had the given condition.

| **Category** | **Condition** | **Count** |
| --- | --- | --- |
| Rhythms | Aberrant conduction | 1 |
| Rhythms | Atrial fibrillation | 15 |
| Rhythms | Atrial flutter, typical | 3 |
| Rhythms | Irregular sinus rhythm | 2 |
| Rhythms | Sinus arrhythmia | 8 |
| Rhythms | Sinus bradycardia | 25 |
| Rhythms | Sinus rhythm | 143 |
| Rhythms | Sinus tachycardia | 4 |
| Rhythms | Wandering atrial pacemaker | 3 |
| Electric axis of the heart | Electric axis of the heart: horizontal | 20 |
| Electric axis of the heart | Electric axis of the heart: left axis deviation | 66 |
| Electric axis of the heart | Electric axis of the heart: normal | 75 |
| Electric axis of the heart | Electric axis of the heart: right axis deviation | 3 |
| Electric axis of the heart | Electric axis of the heart: vertical | 26 |
| Conduction abnormalities | Complete left bundle branch block | 4 |
| Conduction abnormalities | Complete right bundle branch block | 4 |
| Conduction abnormalities | I degree av block | 10 |
| Conduction abnormalities | Iii degree av-block | 5 |
| Conduction abnormalities | Incomplete left bundle branch block | 6 |
| Conduction abnormalities | Incomplete right bundle branch block | 29 |
| Conduction abnormalities | Left anterior hemiblock | 16 |
| Conduction abnormalities | Non-specific intravintricular conduction delay | 4 |
| Conduction abnormalities | Sinoatrial blockade, undefined | 1 |
| Extrasystolies | Atrial extrasystole, type: allorhythmic pattern | 1 |
| Extrasystolies | Atrial extrasystole, type: bigemini | 1 |
| Extrasystolies | Atrial extrasystole, type: quadrigemini | 1 |
| Extrasystolies | Atrial extrasystole, type: single pac | 4 |
| Extrasystolies | Atrial extrasystole: left atrial | 2 |
| Extrasystolies | Atrial extrasystole: low atrial | 1 |
| Extrasystolies | Atrial extrasystole: sa-nodal extrasystole | 3 |
| Extrasystolies | Atrial extrasystole: undefined | 2 |
| Extrasystolies | Ventricular extrasystole, localisation: ivs, iddle part | 1 |
| Extrasystolies | Ventricular extrasystole, localisation: lv, undefined | 1 |
| Extrasystolies | Ventricular extrasystole, localisation: lvot, lvs | 2 |
| Extrasystolies | Ventricular extrasystole, localisation: rvot, anterior wall | 3 |
| Extrasystolies | Ventricular extrasystole, localisation: rvot, antero-septal part | 1 |
| Extrasystolies | Ventricular extrasystole, morphology: polymorphic | 2 |
| Extrasystolies | Ventricular extrasystole, type: couplet | 2 |
| Extrasystolies | Ventricular extrasystole, type: intercalary pvc | 2 |
| Extrasystolies | Ventricular extrasystole, type: single pvc | 6 |
| Hypertrophies | Left atrial hypertrophy | 102 |
| Hypertrophies | Left atrial overload | 11 |
| Hypertrophies | Left ventricular hypertrophy | 108 |
| Hypertrophies | Left ventricular overload | 11 |
| Hypertrophies | Right atrial hypertrophy | 1 |
| Hypertrophies | Right atrial overload | 17 |
| Hypertrophies | Right ventricular hypertrophy | 3 |
| Cardiac pacing | Bipolar ventricular pacing | 2 |
| Cardiac pacing | Biventricular pacing | 1 |
| Cardiac pacing | P-synchrony | 2 |
| Cardiac pacing | Pacemaker presence, undefined | 9 |
| Cardiac pacing | Unipolar atrial pacing | 1 |
| Cardiac pacing | Unipolar ventricular pacing | 6 |
| Ischemia | Ischemia: anterior wall | 5 |
| Ischemia | Ischemia: apical | 6 |
| Ischemia | Ischemia: inferior wall | 10 |
| Ischemia | Ischemia: lateral wall | 8 |
| Ischemia | Ischemia: posterior wall | 2 |
| Ischemia | Ischemia: septal | 4 |
| Ischemia | Scar formation: anterior wall | 8 |
| Ischemia | Scar formation: apical | 5 |
| Ischemia | Scar formation: inferior wall | 3 |
| Ischemia | Scar formation: lateral wall | 3 |
| Ischemia | Scar formation: posterior wall | 6 |
| Ischemia | Scar formation: septal | 9 |
| Ischemia | Stemi: anterior wall | 8 |
| Ischemia | Stemi: apical | 5 |
| Ischemia | Stemi: inferior wall | 1 |
| Ischemia | Stemi: lateral wall | 7 |
| Ischemia | Stemi: septal | 8 |
| Ischemia | Undefined ischemia/scar/supp.nstemi: anterior wall | 12 |
| Ischemia | Undefined ischemia/scar/supp.nstemi: apical | 11 |
| Ischemia | Undefined ischemia/scar/supp.nstemi: inferior wall | 3 |
| Ischemia | Undefined ischemia/scar/supp.nstemi: lateral wall | 16 |
| Ischemia | Undefined ischemia/scar/supp.nstemi: posterior wall | 4 |
| Ischemia | Undefined ischemia/scar/supp.nstemi: septal | 5 |
| Non-specific repolarization | Non-specific repolarization abnormalities: anterior wall | 18 |
| abnormalities |  |  |
| Non-specific repolarization | Non-specific repolarization abnormalities: apical | 11 |
| abnormalities |  |  |
| Non-specific repolarization | Non-specific repolarization abnormalities: inferior wall | 19 |
| abnormalities |  |  |
| Non-specific repolarization | Non-specific repolarization abnormalities: lateral wall | 13 |
| abnormalities |  |  |
| Non-specific repolarization | Non-specific repolarization abnormalities: posterior wall | 9 |
| abnormalities |  |  |
| Non-specific repolarization | Non-specific repolarization abnormalities: septal | 15 |
| abnormalities |  |  |
| Other states | Early repolarization syndrome | 9 |

# **Table S3**

**All conditions available in the additional information for the Physionet Challenge 2021 dataset.** The first column shows the name of the condition; the second column the abbreviation (Abbr.) used in this work and the final column shows the SNOMED CT Code. Please note: The names of the conditions are supplied as in the original dataset even though these contain some typos.

| **Condition** | **Abbr.** | **SNOMED CT** |
| --- | --- | --- |
| 1st degree av block | IAVB | 270492004 |
| 2nd degree av block | IIAVB | 195042002 |
| abnormal QRS | abQRS | 164951009 |
| accelerated atrial escape rhythm | AAR | 233892002 |
| accelerated idioventricular rhythm | AIVR | 61277005 |
| accelerated junctional rhythm | AJR | 426664006 |
| acute myocardial infarction | AMI | 57054005 |
| acute myocardial ischemia | AMIs | 413444003 |
| anterior ischemia | AnMIs | 426434006 |
| anterior myocardial infarction | AnMI | 54329005 |
| atrial bigeminy | AB | 251173003 |
| atrial escape beat | AED | 251187003 |
| atrial fibrillation | AF | 164889003 |
| atrial fibrillation and flutter | AFAFL | 195080001 |
| atrial flutter | AFL | 164890007 |
| atrial hypertrophy | AH | 195126007 |
| atrial pacing pattern | AP | 251268003 |
| atrial rhythm | ARH | 106068003 |
| atrial tachycardia | ATach | 713422000 |
| atrioventricular node reentrant tachycardia | AVNRT | 251166008 |
| atrioventricular dissociation | AVD | 50799005 |
| atrioventricular junctional rhythm | AVJR | 29320008 |
| atrioventricular reentrant tachycardia | AVRT | 233897008 |
| av block | AVB | 233917008 |
| blocked premature atrial contraction | BPAC | 251170000 |
| brady tachy syndrome | BTS | 74615001 |
| bradycardia | Brady | 426627000 |
| Brugada syndrome | BRU | 418818005 |
| bundle branch block | BBB | 6374002 |
| cardiac dysrhythmia | CD | 698247007 |
| chronic atrial fibrillation | CAF | 426749004 |
| chronic myocardial ischemia | CMI | 413844008 |
| clockwise or counterclockwise vectorcardiographic loop | CVCL/ CCVCL | 61721007 |
| clockwise rotation | CR | 251198002 |
| complete heart block | CHB | 27885002 |
| complete left bundle branch block | CLBBB | 733534002 |
| complete right bundle branch block | CRBBB | 713427006 |
| congenital incomplete atrioventricular heart block | CIAHB | 204384007 |
| coronary heart disease | CHD | 53741008 |
| counterclockwise rotation | CCR | 251199005 |
| decreased qt interval | SQT | 77867006 |
| diffuse intraventricular block | DIB | 82226007 |
| early repolarization | ERe | 428417006 |
| fqrs wave | FQRS | 164942001 |
| fusion beats | FB | 13640000 |
| heart failure | HF | 84114007 |
| heart valve disorder | HVD | 368009 |
| high t-voltage | HTV | 251259000 |
| idioventricular rhythm | IR | 49260003 |
| incomplete left bundle branch block | ILBBB | 251120003 |
| incomplete right bundle branch block | IRBBB | 713426002 |
| indeterminate cardiac axis | ICA | 251200008 |
| inferior ischemia | IIs | 425419005 |
| inferior ST segment depression | ISTD | 704997005 |
| junctional escape | JE | 426995002 |
| junctional premature complex | JPC | 251164006 |
| junctional tachycardia | JTach | 426648003 |
| lateral ischemia | LIs | 425623009 |
| left anterior fascicular block | LAnFB | 445118002 |
| left atrial abnormality | LAA | 253352002 |
| left atrial enlargement | LAE | 6.77E+13 |
| left atrial hypertrophy | LAH | 446813000 |
| left axis deviation | LAD | 39732003 |
| left bundle branch block | LBBB | 164909002 |
| left posterior fascicular block | LPFB | 445211001 |
| left ventricular high voltage | LVHV | 55827005 |
| left ventricular hypertrophy | LVH | 164873001 |
| left ventricular strain | LVS | 370365005 |
| low qrs voltages | LQRSV | 251146004 |
| mobitz type i wenckebach atrioventricular block | MoI | 54016002 |
| mobitz type II atrioventricular block | IIAVBII | 426183003 |
| myocardial infarction | MI | 164865005 |
| myocardial ischemia | MIs | 164861001 |
| nonspecific intraventricular conduction disorder | NSIVCB | 698252002 |
| nonspecific st t abnormality | NSSTTA | 428750005 |
| old myocardial infarction | OldMI | 164867002 |
| p wave change | PWC | 164912004 |
| pacing rhythm | PR | 10370003 |
| paired ventricular premature complexes | VPVC | 251182009 |
| paroxysmal atrial fibrillation | PAF | 282825002 |
| paroxysmal supraventricular tachycardia | PSVT | 67198005 |
| paroxysmal ventricular tachycardia | PVT | 425856008 |
| poor R wave Progression | PRWP | 365413008 |
| premature atrial contraction | PAC | 284470004 |
| premature ventricular contractions | PVC | 427172004 |
| prolonged P wave | PPW | 251205003 |
| prolonged pr interval | LPR | 164947007 |
| prolonged qt interval | LQT | 111975006 |
| qwave abnormal | QAb | 164917005 |
| r wave abnormal | RAb | 164921003 |
| rapid atrial fibrillation | RAF | 314208002 |
| right atrial high voltage | RAHV | 6.78E+13 |
| right atrial abnormality | RAAb | 253339007 |
| right atrial hypertrophy | RAH | 446358003 |
| right axis deviation | RAD | 47665007 |
| right bundle branch block | RBBB | 59118001 |
| right ventricular hypertrophy | RVH | 89792004 |
| s t changes | STC | 55930002 |
| shortened pr interval | SPRI | 49578007 |
| sinoatrial block | SAB | 65778007 |
| sinus arrest | SARR | 5609005 |
| sinus arrhythmia | SA | 427393009 |
| sinus atrium to atrial wandering rhythm | SAAWR | 17366009 |
| sinus bradycardia | SB | 426177001 |
| sinus node dysfunction | SND | 60423000 |
| sinus rhythm | NSR | 426783006 |
| sinus tachycardia | STach | 427084000 |
| st depression | STD | 429622005 |
| st elevation | STE | 164931005 |
| st interval abnormal | STIAb | 164930006 |
| supraventricular bigeminy | SVB | 251168009 |
| supraventricular premature beats | SVPB | 63593006 |
| supraventricular tachycardia | SVT | 426761007 |
| suspect arm ecg leads reversed | ALR | 251139008 |
| t wave abnormal | TAb | 164934002 |
| t wave inversion | TInv | 59931005 |
| tall p wave | TPW | 251223006 |
| transient ischemic attack | TIA | 266257000 |
| u wave abnormal | UAb | 164937009 |
| ventricular bigeminy | VBig | 11157007 |
| ventricular ectopics | VEB | 164884008 |
| ventricular escape beat | VEsB | 75532003 |
| ventricular escape rhythm | VEsR | 81898007 |
| ventricular fibrillation | VF | 164896001 |
| ventricular flutter | VFL | 111288001 |
| ventricular hypertrophy | VH | 266249003 |
| ventricular pacing pattern | VPP | 251266004 |
| ventricular pre excitation | VPEx | 195060002 |
| ventricular premature beats | VPB | 17338001 |
| ventricular tachycardia | VTach | 164895002 |
| ventricular trigeminy | VTrig | 251180001 |
| wandering atrial pacemaker | WAP | 195101003 |
| wolff parkinson white pattern | WPW | 74390002 |

# **Table S4**

**Hyperparameters considered in the model optimization experiments.**

| **Hyperparameter** | **Options** |
| --- | --- |
| activation function | ReLU, GELU |
| batch size | 64 |
| d_model | 32, 64, 128, 256 |
| dim_feedforward | 128, 256, 512 |
| dropout | 0.1, 0.2 |
| epochs | 128 |
| kernel size | 9, 17, 33 |
| Initial learning rate | 0.0001, 0.0005, 0.001, 0.005, 0.01 |
| num_heads | 4, 8, 16 |
| num_layers | 2, 3, 4 |
| optimizer | RAdam |
| padding | 4, 8, 16 |
| pos_encoding | fixed, learnable |
| stride | 1, 2, 4, 8, 16 |

# **Table S5**

**Best performing models on the respective evaluation sets. For each model the selected hyperparameter combination is shown along with the best and the last MAE and loss.**

| **Hyperparameter** | **Fold 0** | **Fold 1** | **Fold 2** | **Fold 3** | **Fold 4** |
| --- | --- | --- | --- | --- | --- |
| activation function | ReLU | ReLU | GELU | ReLU | ReLU |
| d_model | 64 | 64 | 64 | 32 | 128 |
| dim_forward | 128 | 512 | 128 | 256 | 256 |
| dropout | 0.1 | 0.1 | 0.2 | 0.1 | 0.1 |
| kernel_size | 33 | 33 | 33 | 33 | 33 |
| stride | 8 | 8 | 8 | 16 | 4 |
| padding | 4 | 16 | 8 | 8 | 16 |
| learning_rate | 0.0005 | 0.001 | 0.0005 | 0.0005 | 0.0001 |
| num_heads | 16 | 4 | 16 | 8 | 4 |
| num_layers | 4 | 4 | 4 | 2 | 3 |
| pos_encoding | fixed | fixed | learnable | fixed | learnable |
| best MAE | 8.18 | 8.72 | 9.03 | 9.47 | 8.35 |
| best loss | 113.78 | 128.51 | 145.07 | 351.8 | 118.36 |
| last MAE | 8.31 | 9.06 | 8.95 | 9.52 | 8.58 |
| last loss | 120.59 | 136.85 | 148.09 | 353.68 | 125.08 |

# **Table S6**

**Pairwise log rank statistics for the Cox Model.**

| **Dataset** | **N at risk (low)** | **N at risk (high)** | **N Events (low)** | **N Events (high)** | **Test Statistic** | **p** |
| --- | --- | --- | --- | --- | --- | --- |
| Code-15 | 53869 | 46097 | 3196 | 996 | 830.9993 | <0.0001 |
| SaMiTrop | 291 | 497 | 28 | 30 | 3.4829 | 0.0620 |

# **Table S7**

**Characteristics of ECGs included versus excluded from the explainability analysis.**
One row per dataset; final row ('Overall') pools all datasets. Age: mean ± SD; sex: n (%). P-values: Welch's t-test (age), χ² test (sex), included vs. all excluded.

| **Dataset** | **N_total_** | **N_included_** | **N_excluded (delineation)_** | **N_excluded  (out-of-range)_** | **% excluded** | **Age included  (mean ± SD)** | **Age excluded  (mean ± SD)** | **p (age)** | **Male included** | **Male excluded** | **p (sex)** |
| --- | --- | --- | --- | --- | --- | --- | --- | --- | --- | --- | --- |
| CODE-15 | 345779 | 291166 | 1561 | 53052 | 15.8% | 52.7 ± 19.6 | 56.2 ± 20.0 | <0.001 | 111,306 (38.2%) | 27,897 (51.1%) | <0.001 |
| SaMiTrop | 1631 | 1217 | 26 | 388 | 25.4% | 58.8 ± 12.5 | 61.2 ± 13.4 | 0.001 | 368 (30.2%) | 166 (40.1%) | <0.001 |
| CPSC | 6868 | 5392 | 1 | 1475 | 21.5% | 59.9 ± 19.3 | 61.5 ± 18.0 | 0.003 | 2,827 (52.4%) | 870 (58.9%) | <0.001 |
| CPSC extra | 3450 | 2668 | 15 | 767 | 22.7% | 63.9 ± 15.3 | 63.4 ± 16.0 | 0.462 | 1,393 (52.2%) | 450 (57.5%) | 0.010 |
| INCART | 74 | 45 | 1 | 28 | 39.2% | 58.7 ± 12.8 | 51.8 ± 14.6 | 0.044 | 26 (57.8%) | 14 (48.3%) | 0.574 |
| PTB | 507 | 392 | 4 | 111 | 22.7% | 56.2 ± 13.8 | 56.5 ± 14.8 | 0.889 | 285 (72.7%) | 86 (74.8%) | 0.747 |
| PTB-XL | 21544 | 17398 | 39 | 4107 | 19.2% | 59.2 ± 16.9 | 61.1 ± 16.2 | <0.001 | 8,855 (50.9%) | 2,456 (59.2%) | <0.001 |
| Georgia | 10267 | 7842 | 28 | 2397 | 23.6% | 60.6 ± 15.4 | 60.1 ± 15.6 | 0.143 | 4,143 (52.8%) | 1,374 (56.7%) | 0.001 |
| Ningbo | 34602 | 25571 | 237 | 8794 | 26.1% | 57.3 ± 20.4 | 60.3 ± 18.2 | <0.001 | 14,003 (54.8%) | 5,550 (61.5%) | <0.001 |
| Shaoxing | 10247 | 7240 | 38 | 2969 | 29.3% | 60.2 ± 17.1 | 59.9 ± 16.9 | 0.544 | 3,922 (54.2%) | 1,811 (60.2%) | <0.001 |
| LUDB | 200 | 172 | 4 | 24 | 14.0% | 50.5 ± 19.3 | 61.0 ± 16.5 | 0.004 | 94 (54.7%) | 21 (75.0%) | 0.070 |
| LEAD | 14462 | 11376 | 9 | 3077 | 21.3% | 37.3 ± 21.3 | 41.7 ± 19.1 | <0.001 | — | — | — |
| **Overall** | **449631** | **370479** | **1963** | **77189** | **17.6%** | **53.4 ± 19.8** | **56.8 ± 19.6** | **<0.001** | **147,222 (39.7%)** | **40,695 (51.4%)** | **<0.001** |

# **Table S8**

**ECG findings and comorbidities of included versus excluded recordings, by dataset.**
Values: n (%). P-values: χ² test (included vs. all excluded). Empty cells indicate the finding was not annotated for that dataset. Datasets without any annotation metadata are omitted.

| **Characteristic** | **Included, n (%)** | **Excl. – Delin. failure, n (%)** | **Excl. – Out-of-range, n (%)** | **p (Incl. vs all Excl.)** |
| --- | --- | --- | --- | --- |
| **CODE-15 (Included: 291,166 · Delin. failure: 1,561 · Out-of-range: 53,052)** |  |  |  |  |
| Sinus Bradycardia | 2,789 (1.0%) | 11 (0.7%) | 2,805 (5.3%) | <0.001 |
| Atrioventricular Block | 4,126 (1.4%) | 21 (1.3%) | 1,569 (3.0%) | <0.001 |
| LBBB | 3,799 (1.3%) | 42 (2.7%) | 2,185 (4.1%) | <0.001 |
| RBBB | 7,326 (2.5%) | 79 (5.1%) | 2,267 (4.3%) | <0.001 |
| Atrial Fibrillation | 4,688 (1.6%) | 31 (2.0%) | 2,314 (4.4%) | <0.001 |
| Death | 6,585 (2.3%) | 78 (5.0%) | 1,678 (3.2%) | <0.001 |
| **SaMiTrop (Included: 1,217 · Delin. failure: 26 · Out-of-range: 388)** |  |  |  |  |
| Death | 59 (4.8%) | 6 (23.1%) | 39 (10.1%) | <0.001 |
| **CPSC (Included: 5,392 · Delin. failure: 1 · Out-of-range: 1,475)** |  |  |  |  |
| Sinus Arrhythmia | 0 (0.0%) | 0 (0.0%) | 0 (0.0%) | — |
| Normal Sinus Rhythm | 815 (15.1%) | 0 (0.0%) | 100 (6.8%) | <0.001 |
| Early Repolarization | 0 (0.0%) | 0 (0.0%) | 0 (0.0%) | — |
| Sinus Bradycardia | 0 (0.0%) | 0 (0.0%) | 0 (0.0%) | — |
| ST-T Abnormality | 0 (0.0%) | 0 (0.0%) | 0 (0.0%) | — |
| QRS Complex Abnormality | 0 (0.0%) | 0 (0.0%) | 0 (0.0%) | — |
| Poor R-wave Progression | 0 (0.0%) | 0 (0.0%) | 0 (0.0%) | — |
| Q-wave Abnormality | 0 (0.0%) | 0 (0.0%) | 0 (0.0%) | — |
| Intraventricular Conduction Block | 0 (0.0%) | 0 (0.0%) | 0 (0.0%) | — |
| Premature Ventricular Contraction | 0 (0.0%) | 0 (0.0%) | 0 (0.0%) | — |
| Left Ventricular Hypertrophy | 0 (0.0%) | 0 (0.0%) | 0 (0.0%) | — |
| ST Segment Depression | 749 (13.9%) | 0 (0.0%) | 119 (8.1%) | <0.001 |
| Premature Atrial Contraction | 468 (8.7%) | 0 (0.0%) | 147 (10.0%) | 0.140 |
| Myocardial Ischemia | 0 (0.0%) | 0 (0.0%) | 0 (0.0%) | — |
| Left Anterior Fascicular Block | 0 (0.0%) | 0 (0.0%) | 0 (0.0%) | — |
| First-degree AV block | 0 (0.0%) | 0 (0.0%) | 0 (0.0%) | — |
| Left Ventricular High Voltage | 0 (0.0%) | 0 (0.0%) | 0 (0.0%) | — |
| Atrioventricular Block | 601 (11.1%) | 1 (100.0%) | 120 (8.1%) | 0.001 |
| Myocardial Infarction | 0 (0.0%) | 0 (0.0%) | 0 (0.0%) | — |
| T-wave Inversion | 0 (0.0%) | 0 (0.0%) | 0 (0.0%) | — |
| CRBBB | 0 (0.0%) | 0 (0.0%) | 0 (0.0%) | — |
| LBBB | 134 (2.5%) | 0 (0.0%) | 102 (6.9%) | <0.001 |
| RBBB | 1,522 (28.2%) | 0 (0.0%) | 332 (22.5%) | <0.001 |
| ST Segment Change | 0 (0.0%) | 0 (0.0%) | 0 (0.0%) | — |
| T-wave Abnormality | 0 (0.0%) | 0 (0.0%) | 0 (0.0%) | — |
| Left Axis Deviation | 0 (0.0%) | 0 (0.0%) | 0 (0.0%) | — |
| Atrial Fibrillation | 872 (16.2%) | 0 (0.0%) | 348 (23.6%) | <0.001 |
| Atrial Flutter | 0 (0.0%) | 0 (0.0%) | 0 (0.0%) | — |
| **CPSC extra (Included: 2,668 · Delin. failure: 15 · Out-of-range: 767)** |  |  |  |  |
| Sinus Arrhythmia | 10 (0.4%) | 0 (0.0%) | 1 (0.1%) | 0.474 |
| Normal Sinus Rhythm | 4 (0.1%) | 0 (0.0%) | 0 (0.0%) | 0.627 |
| Early Repolarization | 0 (0.0%) | 0 (0.0%) | 0 (0.0%) | — |
| Sinus Bradycardia | 28 (1.0%) | 0 (0.0%) | 17 (2.2%) | 0.024 |
| ST-T Abnormality | 1,017 (38.1%) | 1 (6.7%) | 270 (35.2%) | 0.086 |
| QRS Complex Abnormality | 0 (0.0%) | 0 (0.0%) | 0 (0.0%) | — |
| Poor R-wave Progression | 0 (0.0%) | 0 (0.0%) | 0 (0.0%) | — |
| Q-wave Abnormality | 0 (0.0%) | 0 (0.0%) | 1 (0.1%) | 0.514 |
| Intraventricular Conduction Block | 3 (0.1%) | 1 (6.7%) | 0 (0.0%) | 1.000 |
| Premature Ventricular Contraction | 130 (4.9%) | 0 (0.0%) | 58 (7.6%) | 0.008 |
| Left Ventricular Hypertrophy | 111 (4.2%) | 1 (6.7%) | 46 (6.0%) | 0.038 |
| ST Segment Depression | 43 (1.6%) | 0 (0.0%) | 14 (1.8%) | 0.853 |
| Premature Atrial Contraction | 54 (2.0%) | 0 (0.0%) | 19 (2.5%) | 0.581 |
| Myocardial Ischemia | 318 (11.9%) | 2 (13.3%) | 64 (8.3%) | 0.008 |
| Left Anterior Fascicular Block | 0 (0.0%) | 0 (0.0%) | 0 (0.0%) | — |
| First-degree AV block | 2 (0.1%) | 0 (0.0%) | 1 (0.1%) | 1.000 |
| Left Ventricular High Voltage | 0 (0.0%) | 0 (0.0%) | 0 (0.0%) | — |
| Atrioventricular Block | 76 (2.8%) | 2 (13.3%) | 28 (3.7%) | 0.197 |
| Myocardial Infarction | 300 (11.2%) | 7 (46.7%) | 67 (8.7%) | 0.179 |
| T-wave Inversion | 5 (0.2%) | 0 (0.0%) | 0 (0.0%) | 0.498 |
| CRBBB | 74 (2.8%) | 2 (13.3%) | 37 (4.8%) | 0.003 |
| LBBB | 34 (1.3%) | 1 (6.7%) | 3 (0.4%) | 0.109 |
| RBBB | 0 (0.0%) | 0 (0.0%) | 1 (0.1%) | 0.514 |
| ST Segment Change | 1 (0.0%) | 0 (0.0%) | 0 (0.0%) | 1.000 |
| T-wave Abnormality | 18 (0.7%) | 0 (0.0%) | 4 (0.5%) | 0.804 |
| Left Axis Deviation | 0 (0.0%) | 0 (0.0%) | 0 (0.0%) | — |
| Atrial Fibrillation | 99 (3.7%) | 0 (0.0%) | 54 (7.0%) | <0.001 |
| Atrial Flutter | 26 (1.0%) | 1 (6.7%) | 27 (3.5%) | <0.001 |
| **INCART (Included: 45 · Delin. failure: 1 · Out-of-range: 28)** |  |  |  |  |
| Sinus Arrhythmia | 1 (2.2%) | 0 (0.0%) | 1 (3.6%) | 1.000 |
| Normal Sinus Rhythm | 0 (0.0%) | 0 (0.0%) | 0 (0.0%) | — |
| Early Repolarization | 0 (0.0%) | 0 (0.0%) | 0 (0.0%) | — |
| Sinus Bradycardia | 0 (0.0%) | 0 (0.0%) | 0 (0.0%) | — |
| ST-T Abnormality | 0 (0.0%) | 0 (0.0%) | 0 (0.0%) | — |
| QRS Complex Abnormality | 0 (0.0%) | 0 (0.0%) | 0 (0.0%) | — |
| Poor R-wave Progression | 0 (0.0%) | 0 (0.0%) | 0 (0.0%) | — |
| Q-wave Abnormality | 0 (0.0%) | 0 (0.0%) | 0 (0.0%) | — |
| Intraventricular Conduction Block | 1 (2.2%) | 0 (0.0%) | 0 (0.0%) | 1.000 |
| Premature Ventricular Contraction | 0 (0.0%) | 0 (0.0%) | 0 (0.0%) | — |
| Left Ventricular Hypertrophy | 6 (13.3%) | 0 (0.0%) | 4 (14.3%) | 1.000 |
| ST Segment Depression | 4 (8.9%) | 0 (0.0%) | 0 (0.0%) | 0.261 |
| Premature Atrial Contraction | 3 (6.7%) | 0 (0.0%) | 0 (0.0%) | 0.415 |
| Myocardial Ischemia | 0 (0.0%) | 0 (0.0%) | 0 (0.0%) | — |
| Left Anterior Fascicular Block | 0 (0.0%) | 0 (0.0%) | 0 (0.0%) | — |
| First-degree AV block | 0 (0.0%) | 0 (0.0%) | 0 (0.0%) | — |
| Left Ventricular High Voltage | 0 (0.0%) | 0 (0.0%) | 0 (0.0%) | — |
| Atrioventricular Block | 0 (0.0%) | 0 (0.0%) | 0 (0.0%) | — |
| Myocardial Infarction | 6 (13.3%) | 1 (100.0%) | 2 (7.1%) | 0.984 |
| T-wave Inversion | 1 (2.2%) | 0 (0.0%) | 0 (0.0%) | 1.000 |
| CRBBB | 0 (0.0%) | 0 (0.0%) | 0 (0.0%) | — |
| LBBB | 0 (0.0%) | 0 (0.0%) | 0 (0.0%) | — |
| RBBB | 2 (4.4%) | 0 (0.0%) | 0 (0.0%) | 0.677 |
| ST Segment Change | 0 (0.0%) | 0 (0.0%) | 0 (0.0%) | — |
| T-wave Abnormality | 0 (0.0%) | 0 (0.0%) | 0 (0.0%) | — |
| Left Axis Deviation | 0 (0.0%) | 0 (0.0%) | 0 (0.0%) | — |
| Atrial Fibrillation | 2 (4.4%) | 0 (0.0%) | 0 (0.0%) | 0.677 |
| Atrial Flutter | 0 (0.0%) | 0 (0.0%) | 0 (0.0%) | — |
| **PTB (Included: 392 · Delin. failure: 4 · Out-of-range: 111)** |  |  |  |  |
| Sinus Arrhythmia | 0 (0.0%) | 0 (0.0%) | 0 (0.0%) | — |
| Normal Sinus Rhythm | 59 (15.1%) | 0 (0.0%) | 14 (12.6%) | 0.534 |
| Early Repolarization | 0 (0.0%) | 0 (0.0%) | 0 (0.0%) | — |
| Sinus Bradycardia | 0 (0.0%) | 0 (0.0%) | 0 (0.0%) | — |
| ST-T Abnormality | 0 (0.0%) | 0 (0.0%) | 0 (0.0%) | — |
| QRS Complex Abnormality | 0 (0.0%) | 0 (0.0%) | 0 (0.0%) | — |
| Poor R-wave Progression | 0 (0.0%) | 0 (0.0%) | 0 (0.0%) | — |
| Q-wave Abnormality | 0 (0.0%) | 0 (0.0%) | 0 (0.0%) | — |
| Intraventricular Conduction Block | 0 (0.0%) | 0 (0.0%) | 0 (0.0%) | — |
| Premature Ventricular Contraction | 0 (0.0%) | 0 (0.0%) | 0 (0.0%) | — |
| Left Ventricular Hypertrophy | 0 (0.0%) | 0 (0.0%) | 0 (0.0%) | — |
| ST Segment Depression | 0 (0.0%) | 0 (0.0%) | 0 (0.0%) | — |
| Premature Atrial Contraction | 0 (0.0%) | 0 (0.0%) | 0 (0.0%) | — |
| Myocardial Ischemia | 0 (0.0%) | 0 (0.0%) | 0 (0.0%) | — |
| Left Anterior Fascicular Block | 0 (0.0%) | 0 (0.0%) | 0 (0.0%) | — |
| First-degree AV block | 0 (0.0%) | 0 (0.0%) | 0 (0.0%) | — |
| Left Ventricular High Voltage | 0 (0.0%) | 0 (0.0%) | 0 (0.0%) | — |
| Atrioventricular Block | 0 (0.0%) | 0 (0.0%) | 0 (0.0%) | — |
| Myocardial Infarction | 293 (74.7%) | 1 (25.0%) | 74 (66.7%) | 0.058 |
| T-wave Inversion | 0 (0.0%) | 0 (0.0%) | 0 (0.0%) | — |
| CRBBB | 0 (0.0%) | 0 (0.0%) | 0 (0.0%) | — |
| LBBB | 0 (0.0%) | 0 (0.0%) | 0 (0.0%) | — |
| RBBB | 0 (0.0%) | 0 (0.0%) | 0 (0.0%) | — |
| ST Segment Change | 0 (0.0%) | 0 (0.0%) | 0 (0.0%) | — |
| T-wave Abnormality | 0 (0.0%) | 0 (0.0%) | 0 (0.0%) | — |
| Left Axis Deviation | 0 (0.0%) | 0 (0.0%) | 0 (0.0%) | — |
| Atrial Fibrillation | 10 (2.6%) | 0 (0.0%) | 5 (4.5%) | 0.492 |
| Atrial Flutter | 1 (0.3%) | 0 (0.0%) | 0 (0.0%) | 1.000 |
| **PTB-XL (Included: 17,398 · Delin. failure: 39 · Out-of-range: 4,107)** |  |  |  |  |
| Sinus Arrhythmia | 602 (3.5%) | 1 (2.6%) | 160 (3.9%) | 0.201 |
| Normal Sinus Rhythm | 15,100 (86.8%) | 15 (38.5%) | 2,808 (68.4%) | <0.001 |
| Early Repolarization | 0 (0.0%) | 0 (0.0%) | 0 (0.0%) | — |
| Sinus Bradycardia | 366 (2.1%) | 1 (2.6%) | 267 (6.5%) | <0.001 |
| ST-T Abnormality | 262 (1.5%) | 0 (0.0%) | 104 (2.5%) | <0.001 |
| QRS Complex Abnormality | 2,749 (15.8%) | 15 (38.5%) | 590 (14.4%) | 0.057 |
| Poor R-wave Progression | 0 (0.0%) | 0 (0.0%) | 0 (0.0%) | — |
| Q-wave Abnormality | 451 (2.6%) | 0 (0.0%) | 76 (1.9%) | 0.005 |
| Intraventricular Conduction Block | 612 (3.5%) | 4 (10.3%) | 158 (3.8%) | 0.244 |
| Premature Ventricular Contraction | 0 (0.0%) | 0 (0.0%) | 0 (0.0%) | — |
| Left Ventricular Hypertrophy | 1,737 (10.0%) | 1 (2.6%) | 573 (14.0%) | <0.001 |
| ST Segment Depression | 738 (4.2%) | 0 (0.0%) | 232 (5.6%) | <0.001 |
| Premature Atrial Contraction | 271 (1.6%) | 0 (0.0%) | 116 (2.8%) | <0.001 |
| Myocardial Ischemia | 1,610 (9.3%) | 1 (2.6%) | 507 (12.3%) | <0.001 |
| Left Anterior Fascicular Block | 1,169 (6.7%) | 9 (23.1%) | 390 (9.5%) | <0.001 |
| First-degree AV block | 190 (1.1%) | 13 (33.3%) | 85 (2.1%) | <0.001 |
| Left Ventricular High Voltage | 0 (0.0%) | 0 (0.0%) | 0 (0.0%) | — |
| Atrioventricular Block | 565 (3.2%) | 9 (23.1%) | 198 (4.8%) | <0.001 |
| Myocardial Infarction | 4,105 (23.6%) | 15 (38.5%) | 1,026 (25.0%) | 0.042 |
| T-wave Inversion | 226 (1.3%) | 0 (0.0%) | 59 (1.4%) | 0.580 |
| CRBBB | 397 (2.3%) | 7 (17.9%) | 124 (3.0%) | 0.001 |
| LBBB | 257 (1.5%) | 7 (17.9%) | 243 (5.9%) | <0.001 |
| RBBB | 0 (0.0%) | 0 (0.0%) | 0 (0.0%) | — |
| ST Segment Change | 563 (3.2%) | 0 (0.0%) | 175 (4.3%) | 0.002 |
| T-wave Abnormality | 1,887 (10.8%) | 0 (0.0%) | 407 (9.9%) | 0.057 |
| Left Axis Deviation | 3,927 (22.6%) | 24 (61.5%) | 1,081 (26.3%) | <0.001 |
| Atrial Fibrillation | 950 (5.5%) | 6 (15.4%) | 495 (12.1%) | <0.001 |
| Atrial Flutter | 30 (0.2%) | 5 (12.8%) | 38 (0.9%) | <0.001 |
| **Georgia (Included: 7,842 · Delin. failure: 28 · Out-of-range: 2,397)** |  |  |  |  |
| Sinus Arrhythmia | 382 (4.9%) | 0 (0.0%) | 72 (3.0%) | <0.001 |
| Normal Sinus Rhythm | 1,532 (19.5%) | 0 (0.0%) | 200 (8.3%) | <0.001 |
| Early Repolarization | 100 (1.3%) | 0 (0.0%) | 40 (1.7%) | 0.197 |
| Sinus Bradycardia | 1,000 (12.8%) | 1 (3.6%) | 670 (28.0%) | <0.001 |
| ST-T Abnormality | 1,428 (18.2%) | 0 (0.0%) | 433 (18.1%) | 0.715 |
| QRS Complex Abnormality | 0 (0.0%) | 0 (0.0%) | 0 (0.0%) | — |
| Poor R-wave Progression | 0 (0.0%) | 0 (0.0%) | 0 (0.0%) | — |
| Q-wave Abnormality | 352 (4.5%) | 0 (0.0%) | 103 (4.3%) | 0.654 |
| Intraventricular Conduction Block | 151 (1.9%) | 0 (0.0%) | 52 (2.2%) | 0.553 |
| Premature Ventricular Contraction | 0 (0.0%) | 0 (0.0%) | 0 (0.0%) | — |
| Left Ventricular Hypertrophy | 899 (11.5%) | 0 (0.0%) | 333 (13.9%) | 0.003 |
| ST Segment Depression | 25 (0.3%) | 0 (0.0%) | 13 (0.5%) | 0.177 |
| Premature Atrial Contraction | 459 (5.9%) | 1 (3.6%) | 176 (7.3%) | 0.011 |
| Myocardial Ischemia | 0 (0.0%) | 0 (0.0%) | 0 (0.0%) | — |
| Left Anterior Fascicular Block | 129 (1.6%) | 1 (3.6%) | 47 (2.0%) | 0.309 |
| First-degree AV block | 0 (0.0%) | 0 (0.0%) | 0 (0.0%) | — |
| Left Ventricular High Voltage | 0 (0.0%) | 0 (0.0%) | 0 (0.0%) | — |
| Atrioventricular Block | 577 (7.4%) | 1 (3.6%) | 189 (7.9%) | 0.461 |
| Myocardial Infarction | 6 (0.1%) | 0 (0.0%) | 1 (0.0%) | 0.891 |
| T-wave Inversion | 594 (7.6%) | 2 (7.1%) | 210 (8.8%) | 0.068 |
| CRBBB | 16 (0.2%) | 0 (0.0%) | 12 (0.5%) | 0.029 |
| LBBB | 133 (1.7%) | 5 (17.9%) | 93 (3.9%) | <0.001 |
| RBBB | 412 (5.3%) | 7 (25.0%) | 119 (5.0%) | 0.952 |
| ST Segment Change | 5 (0.1%) | 0 (0.0%) | 1 (0.0%) | 1.000 |
| T-wave Abnormality | 1,729 (22.0%) | 2 (7.1%) | 559 (23.3%) | 0.274 |
| Left Axis Deviation | 676 (8.6%) | 12 (42.9%) | 249 (10.4%) | 0.002 |
| Atrial Fibrillation | 420 (5.4%) | 6 (21.4%) | 142 (5.9%) | 0.175 |
| Atrial Flutter | 117 (1.5%) | 0 (0.0%) | 68 (2.8%) | <0.001 |
| **Ningbo (Included: 25,571 · Delin. failure: 237 · Out-of-range: 8,794)** |  |  |  |  |
| Sinus Arrhythmia | 2,190 (8.6%) | 1 (0.4%) | 355 (4.0%) | <0.001 |
| Normal Sinus Rhythm | 5,775 (22.6%) | 2 (0.8%) | 359 (4.1%) | <0.001 |
| Early Repolarization | 195 (0.8%) | 0 (0.0%) | 148 (1.7%) | <0.001 |
| Sinus Bradycardia | 8,287 (32.4%) | 15 (6.3%) | 4,352 (49.5%) | <0.001 |
| ST-T Abnormality | 0 (0.0%) | 0 (0.0%) | 0 (0.0%) | — |
| QRS Complex Abnormality | 0 (0.0%) | 0 (0.0%) | 0 (0.0%) | — |
| Poor R-wave Progression | 442 (1.7%) | 6 (2.5%) | 188 (2.1%) | 0.012 |
| Q-wave Abnormality | 603 (2.4%) | 13 (5.5%) | 202 (2.3%) | 0.935 |
| Intraventricular Conduction Block | 350 (1.4%) | 0 (0.0%) | 179 (2.0%) | <0.001 |
| Premature Ventricular Contraction | 608 (2.4%) | 5 (2.1%) | 459 (5.2%) | <0.001 |
| Left Ventricular Hypertrophy | 353 (1.4%) | 3 (1.3%) | 267 (3.0%) | <0.001 |
| ST Segment Depression | 761 (3.0%) | 6 (2.5%) | 496 (5.6%) | <0.001 |
| Premature Atrial Contraction | 664 (2.6%) | 6 (2.5%) | 372 (4.2%) | <0.001 |
| Myocardial Ischemia | 0 (0.0%) | 0 (0.0%) | 0 (0.0%) | — |
| Left Anterior Fascicular Block | 227 (0.9%) | 2 (0.8%) | 150 (1.7%) | <0.001 |
| First-degree AV block | 696 (2.7%) | 160 (67.5%) | 323 (3.7%) | <0.001 |
| Left Ventricular High Voltage | 2,809 (11.0%) | 3 (1.3%) | 1,271 (14.5%) | <0.001 |
| Atrioventricular Block | 600 (2.3%) | 3 (1.3%) | 286 (3.3%) | <0.001 |
| Myocardial Infarction | 52 (0.2%) | 1 (0.4%) | 24 (0.3%) | 0.253 |
| T-wave Inversion | 1,853 (7.2%) | 10 (4.2%) | 846 (9.6%) | <0.001 |
| CRBBB | 765 (3.0%) | 6 (2.5%) | 319 (3.6%) | 0.005 |
| LBBB | 17 (0.1%) | 0 (0.0%) | 18 (0.2%) | 0.001 |
| RBBB | 141 (0.6%) | 0 (0.0%) | 54 (0.6%) | 0.670 |
| ST Segment Change | 2,703 (10.6%) | 18 (7.6%) | 1,451 (16.5%) | <0.001 |
| T-wave Abnormality | 3,804 (14.9%) | 12 (5.1%) | 1,314 (14.9%) | 0.669 |
| Left Axis Deviation | 795 (3.1%) | 15 (6.3%) | 345 (3.9%) | <0.001 |
| Atrial Fibrillation | 0 (0.0%) | 0 (0.0%) | 0 (0.0%) | — |
| Atrial Flutter | 5,205 (20.4%) | 100 (42.2%) | 2,260 (25.7%) | <0.001 |
| **Shaoxing (Included: 7,240 · Delin. failure: 38 · Out-of-range: 2,969)** |  |  |  |  |
| Sinus Arrhythmia | 0 (0.0%) | 0 (0.0%) | 0 (0.0%) | — |
| Normal Sinus Rhythm | 1,736 (24.0%) | 0 (0.0%) | 90 (3.0%) | <0.001 |
| Early Repolarization | 14 (0.2%) | 0 (0.0%) | 8 (0.3%) | 0.625 |
| Sinus Bradycardia | 2,564 (35.4%) | 0 (0.0%) | 1,325 (44.6%) | <0.001 |
| ST-T Abnormality | 754 (10.4%) | 0 (0.0%) | 404 (13.6%) | <0.001 |
| QRS Complex Abnormality | 0 (0.0%) | 0 (0.0%) | 0 (0.0%) | — |
| Poor R-wave Progression | 0 (0.0%) | 0 (0.0%) | 0 (0.0%) | — |
| Q-wave Abnormality | 175 (2.4%) | 2 (5.3%) | 58 (2.0%) | 0.220 |
| Intraventricular Conduction Block | 147 (2.0%) | 0 (0.0%) | 88 (3.0%) | 0.007 |
| Premature Ventricular Contraction | 0 (0.0%) | 0 (0.0%) | 0 (0.0%) | — |
| Left Ventricular Hypertrophy | 8 (0.1%) | 0 (0.0%) | 7 (0.2%) | 0.234 |
| ST Segment Depression | 259 (3.6%) | 0 (0.0%) | 143 (4.8%) | 0.006 |
| Premature Atrial Contraction | 155 (2.1%) | 2 (5.3%) | 101 (3.4%) | <0.001 |
| Myocardial Ischemia | 0 (0.0%) | 0 (0.0%) | 0 (0.0%) | — |
| Left Anterior Fascicular Block | 0 (0.0%) | 0 (0.0%) | 0 (0.0%) | — |
| First-degree AV block | 0 (0.0%) | 0 (0.0%) | 0 (0.0%) | — |
| Left Ventricular High Voltage | 860 (11.9%) | 1 (2.6%) | 434 (14.6%) | <0.001 |
| Atrioventricular Block | 187 (2.6%) | 0 (0.0%) | 60 (2.0%) | 0.090 |
| Myocardial Infarction | 21 (0.3%) | 3 (7.9%) | 16 (0.5%) | 0.019 |
| T-wave Inversion | 107 (1.5%) | 0 (0.0%) | 50 (1.7%) | 0.545 |
| CRBBB | 0 (0.0%) | 0 (0.0%) | 0 (0.0%) | — |
| LBBB | 94 (1.3%) | 10 (26.3%) | 101 (3.4%) | <0.001 |
| RBBB | 294 (4.1%) | 5 (13.2%) | 155 (5.2%) | 0.006 |
| ST Segment Change | 0 (0.0%) | 0 (0.0%) | 0 (0.0%) | — |
| T-wave Abnormality | 1,391 (19.2%) | 1 (2.6%) | 484 (16.3%) | <0.001 |
| Left Axis Deviation | 264 (3.6%) | 6 (15.8%) | 112 (3.8%) | 0.536 |
| Atrial Fibrillation | 1,314 (18.1%) | 2 (5.3%) | 464 (15.6%) | 0.001 |
| Atrial Flutter | 266 (3.7%) | 3 (7.9%) | 176 (5.9%) | <0.001 |
| **LUDB (Included: 172 · Delin. failure: 4 · Out-of-range: 24)** |  |  |  |  |
| Healthy ECG | 14 (8.1%) | 0 (0.0%) | 1 (4.2%) | 0.642 |
| Sinus Arrhythmia | 8 (4.7%) | 0 (0.0%) | 0 (0.0%) | 0.519 |
| Normal Sinus Rhythm | 127 (73.8%) | 3 (75.0%) | 13 (54.2%) | 0.112 |
| Early Repolarization | 8 (4.7%) | 0 (0.0%) | 1 (4.2%) | 1.000 |
| Sinus Bradycardia | 19 (11.0%) | 1 (25.0%) | 5 (20.8%) | 0.218 |
| Intraventricular Conduction Block | 3 (1.7%) | 0 (0.0%) | 1 (4.2%) | 1.000 |
| Left Ventricular Hypertrophy | 92 (53.5%) | 1 (25.0%) | 15 (62.5%) | 0.877 |
| Premature Atrial Contraction | 1 (0.6%) | 0 (0.0%) | 1 (4.2%) | 0.652 |
| Atrioventricular Block | 10 (5.8%) | 0 (0.0%) | 0 (0.0%) | 0.400 |
| CRBBB | 4 (2.3%) | 0 (0.0%) | 0 (0.0%) | 0.930 |
| Left Axis Deviation | 57 (33.1%) | 0 (0.0%) | 9 (37.5%) | 1.000 |
| Atrial Fibrillation | 11 (6.4%) | 0 (0.0%) | 4 (16.7%) | 0.279 |
| Atrial Flutter | 2 (1.2%) | 0 (0.0%) | 1 (4.2%) | 0.893 |
| **LEAD (Included: 11,376 · Delin. failure: 9 · Out-of-range: 3,077)** |  |  |  |  |
| Cardiovascular Disease | 1,408 (12.4%) | 4 (44.4%) | 428 (13.9%) | 0.018 |
| Hypertension | 2,013 (17.7%) | 4 (44.4%) | 590 (19.2%) | 0.050 |
| Diabetes Mellitus | 496 (4.4%) | 1 (11.1%) | 116 (3.8%) | 0.130 |
| Hyperlipoproteinemia A | 5,669 (49.8%) | 4 (44.4%) | 1,698 (55.2%) | <0.001 |
| COPD | 646 (5.7%) | 0 (0.0%) | 176 (5.7%) | 1.000 |
| Myocardial Infarction | 202 (1.8%) | 1 (11.1%) | 55 (1.8%) | 0.945 |
| Stroke | 81 (0.7%) | 1 (11.1%) | 28 (0.9%) | 0.240 |
| Angina Pectoris | 128 (1.1%) | 1 (11.1%) | 38 (1.2%) | 0.587 |
| Arrhythmia | 429 (3.8%) | 2 (22.2%) | 160 (5.2%) | <0.001 |
| Kidney Disease | 110 (1.0%) | 2 (22.2%) | 26 (0.8%) | 0.844 |
| Cardiac Insufficiency | 91 (0.8%) | 1 (11.1%) | 31 (1.0%) | 0.246 |
| Peripheral Artery Disease | 155 (1.4%) | 1 (11.1%) | 30 (1.0%) | 0.140 |
| Carotid Artery Calcification | 308 (2.7%) | 0 (0.0%) | 82 (2.7%) | 0.181 |

# **Figure S1**

**Exact split of public datasets into train, validation and test.**

# **Figure S2**

**Multivariate timeseries transformer model used in this work to predict AI-ECG age.** The model uses a 1D Convolutional layer and multiple transformer blocks consisting of several Linear, BatchNorm, ReLU and Dropout layers.

# **Figure S3**

**Histogram of Age for subgroups of LEAD dataset.**

# **Figure S4**

**Correlations of AI-ECG age and chronological age by subgroups of the LEAD cohort.**

# **Figure S5**

**Scatterplots of AI-ECG age and chronological age for all models and datasets.**

# **Figure S6**

**Calibration of Δage across chronological age deciles and sex.** Mean Δage is plotted against age deciles for both the Code-15 (top) and SaMiTrop (bottom) datasets, stratified by sex. The strictly decreasing trend illustrates a standard regression-to-the-mean bias, wherein the model overestimates the age of the youngest quartile and underestimates the age of the oldest quartile. This systematic bias necessitated the use of age-residualized Δage in our supplementary continuous survival models to confirm independent prognostic value.

# **Figure S7**

**Threshold sensitivity analysis of categorical Δage and mortality risk.**

The line plots illustrate the adjusted Hazard Ratios (HR) for all-cause mortality across varying symmetric Δage cutoffs (5, 8, 10, and 15 years) used to define the extreme risk categories. For each evaluated threshold (t), patients were stratified into a higher-risk group (Δage > +t, solid lines) and a lower-risk group (Δage < -t, dashed lines), relative to the normal-aging reference baseline (-t <= Δage <= +t). Analyses were performed independently for the CODE-15 (left) and SaMiTrop (right) cohorts. All hazard ratios were derived using multivariate Cox proportional hazards models adjusted for chronological age and sex. The horizontal dashed line denotes the reference hazard ratio of 1.0.

# **Figure S8**

**Sex and age stratified Saliency Analysis.** (top) correlation of ECG features, (middle) saliency per ECG segment per stratified by sex and age decile.

# **Figure S9**

**Individual dataset analysis.** (top) correlation of ECG features, (middle) saliency per ECG segment, (bottom) Saliency per ECG segment across ECG leads.

# **Figure S10**

**Correlations of comorbidities with AI-ECG age for the public datasets for all four models.**

# **Figure S11**

**Heatmap of the prevalence difference (excluded − included, percentage points) for each ECG finding/comorbidity across datasets, shown separately for the delineation-failure and out-of-range exclusion subgroups; asterisks denote χ² significance.**

# **Figure S12**

**Age distribution across the 12 ECG datasets.**

Each panel shows the histogram of chronological age (5-year bins) for one dataset. Dashed vertical lines indicate the deciles of each dataset's own age distribution. Datasets are ordered by their cross-validation fold membership.

# **Figure S13**

**Age distribution across training, validation, and test sets for each of the five cross-validation folds.**

Each panel shows the histogram of chronological age (5-year bins) for a given split (rows: Train, Validation, Test) and cross-validation fold (columns: Fold 0–4). Dashed vertical lines indicate deciles of the respective split's distribution. Sample sizes are reported in the upper right of each panel. The LEAD cohort is excluded as it served as an external test set independent of the cross-validation.
